# The signaling landscape of insulin-like growth factor 1

**DOI:** 10.1016/j.jbc.2024.108047

**Published:** 2024-12-03

**Authors:** Muhammad Zahid Khan, Jose Luis Zugaza, Ignacio Torres Aleman

**Affiliations:** 1 Achucarro Basque Center for Neuroscience, Leioa, Spain; 2 CIBERNED, Madrid, Spain; 3 Ikerbasque Science Foundation, Bilbao, Spain

**Keywords:** insulin-like growth factor, signaling, insulin receptor substrate 1 (IRS-1), phosphatidylinositide-3 kinase (PI3K), AKT (PKB), Ras protein

## Abstract

The sheer amplitude of biological actions of insulin-like growth factor I (IGF-1) affecting all types of cells in all tissues suggests a vast signaling landscape for this ubiquitous humoral signal. While the canonical signaling pathways primarily involve the Ras/MAPK and PI3K/AKT cascades, the evolutionary conservation of insulin-like peptides (ILPs) and their pathways hints at the potential for novel functions to emerge over time. Indeed, the evolutionary trajectory of ILPs opens the possibility of either novel functions for these two pathways, novel downstream routes, or both. Evidence supporting this notion includes observations of neofunctionalization in bony fishes or crustaceans, and the involvement of ILPs pathways in invertebrate eusociality or in vertebrate bone physiology, respectively. Such evolutionary processes likely contribute to the rich diversity of ILPs signaling observed today. Moreover, the interplay between conserved signaling pathways, such as those implicated in aging (predominantly involving the PI3K-AKT route), and lesser known pathways, such as those mediated by biased G-protein coupled receptors and others even less known, may underpin the context-dependent actions characteristic of ILPs signaling. While canonical IGF-1 signaling is often assumed to account for the intracellular pathways utilized by this growth factor, a comprehensive analysis of all the pathways mediated by the IGF-1 receptor (IGF-1R) remains lacking. This review aims to explore both canonical and non-canonical routes of IGF-1R action across various cell types, offering a detailed examination of the mechanisms underlying IGF-1 signaling and highlighting the significant gaps in our current understanding.

Insulin-like peptides (ILPs) are evolutionarily conserved hormones with an extraordinary variety of functions. Decades ago (1, 2), ILP binding activities were described in microorganisms (see (3) for an update) and, more recently, in viruses (4). However, ILPs have been extensively documented and characterized only in higher species. In invertebrate model systems such as *C. elegans*, ILPs modulate growth, development, germline differentiation, learning/memory, and longevity (5), as well as metabolism (6) or even the microbiome (7). These varieties of actions are mediated through a single receptor and forty ligands (8), some of them with antagonistic properties (9). All these roles are conserved in higher species, and new ones have emerged in parallel with the increase in functional complexity associated with the development of new organs (brain, liver, kidney, etc) or evolving functions (complex behaviors, tissue healing, etc). In parallel, the number of receptors has increased, whereas the ligands have decreased to a point that in vertebrates, each ligand has its preferred single receptor (10).

In line with the highly conserved nature of this family of peptides, their receptors are also conserved along phylogeny (see below). In mammals, the three best-studied receptors are those for insulin and IGF-1, and the mannose-6-phosphate receptor for IGF-2. We will focus this review on the IGF-1 receptor (IGF-1R), but both insulin and IGF-2 (11), can also signal through this receptor, albeit with lower affinity. Moreover, IGF-1R and the insulin receptor (IR), which share high structural homology (12), form functional hybrids that can recognize both ligands and show differential distribution in bodily organs (13). Hybrid receptors may explain the need for both IR and IGF-1R for specific actions of IGF-1 (14), but their physiological significance remains largely unexplored. In addition, while available evidence indicates that hybrid receptors use canonical pathways, their signaling needs additional study.

Although up to six insulin-like growth factor binding proteins (IGFBPs) that bind to IGF-1 and IGF-2, but not insulin, have been identified (15), along with IGFBP proteases (16) we will not explore these in detail in this review. While the IGFBP/IGFBP-protease balance modulates IGF-1 availability, it has only been shown to indirectly affect IGF-1 signaling by influencing IGF-1-related functions (17). Additionally, IGFBPs exhibit IGF-1-independent biological activities, which are constantly being updated (18), representing a distinct area of ILP biology.

In this work, we aim to provide a comprehensive review of IGF-1 signaling in health and disease, including the most recent proposals regarding its context-dependent actions and important areas that require further study, as our current understanding of IGF-1R pathways remains limited.

## The IGF-1 receptor

The transmembrane tyrosine-kinase IGF-1R shares high structural homology with the IR, and probably both arise from gene duplication of a common ancestor (19). This receptor is essential for life (20), is expressed throughout ontogeny (21, 22, 23), and available human single-cell RNA information indicates the highest expression in nerve cells, but all types of cells and tissues express it (https://www.proteinatlas.org/ENSG00000140443-IGF-1R/tissue). These characteristics bespeak the biological relevance of IGF-1 signaling. Intriguingly, downstream IGF-1R signaling is largely shared with insulin (24), showing generally similar gene responses, but with different amplitude (25). At any rate, the mechanisms explaining the distinct functional impact of each hormone are only slowly been unveiled (26, 27), and are not yet entirely clear (28).

IGF-1R is present in the cell membrane and other cell compartments and is composed of two alpha and two beta subunits forming a hetero-tetrameric transmembrane structure bound by disulfide bonds. The alpha subunits face the external space and contain the ligand-binding moieties. The beta subunits are located intramembrane with their C-terminal tails facing the cytoplasm. They contain the tyrosine kinase activity with the corresponding ATP-binding pouch that, upon IGF-1 binding, auto-phosphorylates several tyrosine moieties (29) to initiate downstream signaling.

### IGF-1R ligands

Although each of the three IGF-1R ligands has its own specific receptor and binds to IGF-1R with varying affinities, IGF-1, IGF-1, and insulin share structural homology, including in their receptor-binding regions.

#### IGF-1

IGF-1 is a single-chain polypeptide of 70 amino-acids and has the highest affinity for IGF-1R. Its production is primarily controlled by growth hormone (GH) (30). Most circulating IGF-1 is produced by the liver, with smaller amounts originating from adipose tissue and muscle (31), although nearly all tissues produce this hormone. GH is produced in somatotroph cells in the pituitary gland; its expression and release are mainly regulated by the opposing actions of hypothalamic growth hormone-releasing hormone (GHRH) and somatostatin (32), but IGF-1 also exerts a negative feedback on its production (33). IGF-1 levels are also highly influenced by nutritional intake, which might hold greater significance from an evolutionary standpoint, as the primary signaling pathways triggered by IGF-1 in metazoans were originally activated by nutrient availability (34). Aging is another key factor that regulates IGF-1 levels. As individuals age, there is a decline in the activity of the GH/IGF-1 axis, leading to lower IGF-1 concentrations and diminished functionality of the IGF-1 signaling pathway (35). Gonadal and thyroid hormones also regulate IGF-1. Sex hormones like estradiol promote IGF-1 secretion (36), with normal levels typically stimulating IGF-1 and higher doses inhibiting its production. Thyroid hormones influence the expression and secretion of GH, subsequently affecting the circulating levels of both GH and IGF-1 (37). Other modulators of IGF-1 activity such as exercise (38) and sleep (39) have been also documented, but more work is needed to better define their role in IGF-1 physiology as they exert nuanced actions.

#### IGF-2

This hormone is a single-chain peptide of 67 amino-acids. IGF-2, which is also produced by many tissues throughout the body, binds with slightly lower affinity than IGF-1 to IGF-1R. IGF-2 is a key growth factor during fetal development and is expressed from the paternal allele in mammals (40). The regulation of IGF-2 transcription is intricate and involves multiple levels, starting with its genomic region, which includes the H19 gene, an imprinted control region (ICR), and several enhancers. On the maternal allele, the unmethylated ICR blocks enhancer access to the IGF-2 gene, leading to IGF-2 silencing and promoting H19 expression instead. Conversely, on the paternal allele, the heavily methylated ICR facilitates IGF-2 transcription and results in the silencing of H19 (40). Normal development relies on precise expression levels of IGF-2, and abnormalities in these levels can lead to disorders. External factors such as nutrition, stress, and exposure to toxins can influence the epigenetic marks on the *igf-2* gene. For example, maternal nutrition during pregnancy can affect IGF-2 expression and subsequently impact fetal growth (41). Hormones such as GH, insulin, and thyroid hormones can modulate IGF-2 expression (42, 43).

#### Insulin

This hormone is a double-chained polypeptide connected by two disulfide bonds. This hormone is synthesized by pancreatic β-cells (44) and binds the IGF-1R with 100 lower affinity than IGF-1. Blood glucose modulates insulin secretion *via* glucose transporter (GluT) two in pancreatic β-cells, leading to ATP production, membrane depolarization, calcium influx, and the subsequent release of insulin (45). Other nutrients such as amino acids and fatty acids can also influence insulin secretion (46). Hormones such as glucagon, cortisol, estrogen, leptin, GH, melatonin, and epinephrine modulate insulin secretion, with glucagon (produced by pancreatic α-cells) raising blood glucose levels to counteract insulin, while cortisol and epinephrine modulate insulin release during stress responses (47). Activation of the sympathetic nervous system inhibits insulin secretion during stress or exercise to raise blood glucose levels, while parasympathetic activation stimulates insulin release, especially during digestion (48).

## Canonical IGF-1R signaling

**Figure 1:**
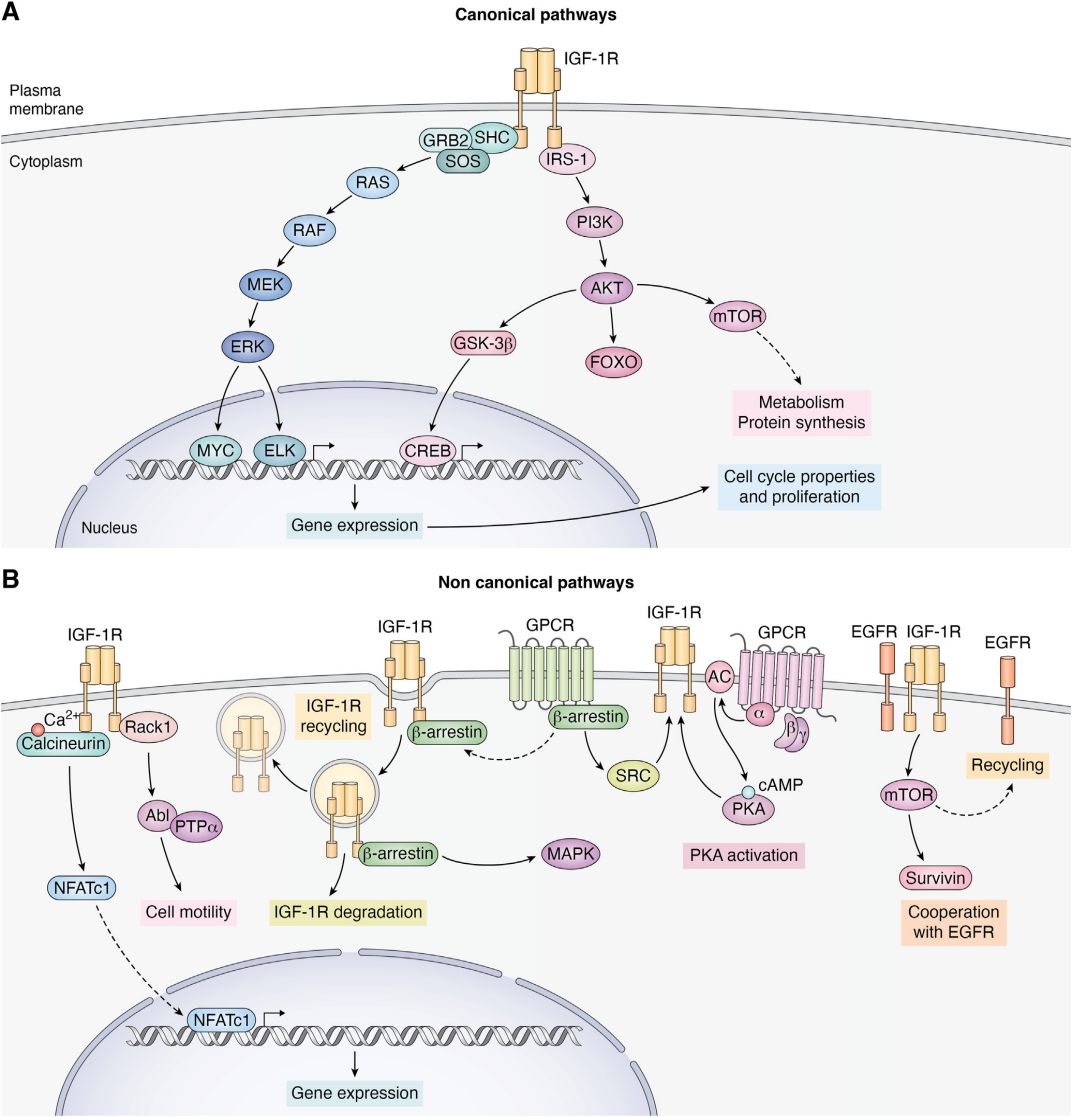
**Signaling Pathways Mediated by IGF-1R.***A*, canonical Pathways: Upon ligand binding, IGF-1R undergoes autophosphorylation on tyrosine residues. This phosphorylation enables IGF-1R to phosphorylate SHC, which recruits the Grb2/SOS tandem to activate Ras. Activated Ras then interacts with and activates RAF, which in turn activates MEK. MEK phosphorylates ERK, which translocates to the nucleus to regulate gene expression through MYC and ELK. This Ras/MAPK pathway is best known to regulate the cell cycle and promotes cell growth. Additionally, IGF-1R phosphorylates IRS-1, which acts as an adaptor protein to activate PI3K. PI3K converts PIP2 to PIP3, a lipid essential for activating AKT. AKT subsequently phosphorylates various substrates, including GSK3β, FOXO, and mTOR. mTOR plays a crucial role in regulating protein synthesis and cell metabolism. *B*, non-Canonical Pathways: we provide several examples that IGF-1R signaling also involves non-canonical pathways that expand cellular control beyond traditional PI3K/AKT or Ras/MAPK pathways. These include interactions with Rack1, effects on calcium/calcineurin/NFAT signaling, interactions with GPCR and arrestin, PKA activation, and cooperation with EGFR. These additional mechanisms allow IGF-1R to modulate a wide range of cellular responses.

Canonical signaling by IGF-1R is shared with IR, has been described in all types of cells and species, and is probably the most commonly used in all cell types (28, 49). As shown in Figure 1*A*, this includes PI3K/AKT and Ras/MAPK pathways. Thus, activated by IGF-1R, PI3K phosphorylates PIP2 to produce PIP3, which recruits AKT to the plasma membrane, where it is phosphorylated and activated (49). Active AKT then phosphorylates several downstream targets such as Bad, Caspase-9, or mTOR (50) (Fig. 1*A*). The outcomes of these phosphorylations vary. Thus, phosphorylation of both Bad and Caspase-9 results in their inactivation, inhibiting in this way apoptosis (51). Conversely, phosphorylation of mTOR activates it, promoting cell survival and growth (50). These combined actions underscore the role of the IGF-1R/PI3K/AKT pathway helping cells to evade apoptosis and continue to proliferate and survive under conditions that might otherwise trigger programmed cell death.

The Ras/MAPK pathway is pivotal for cell proliferation and differentiation (52). The phosphorylated tyrosine residues on IGF-1R serve as docking sites for adaptor proteins such as Shc (Src homolog and collagen domain protein) and IRS-1(Insulin receptor substrate 1) (53). The IRS protein family primarily consists of four closely related members, IRS-1- 4, and two distant ones, IRS5/DOK4 and IRS6/DOK5 (54). Of these, IRS-1 and IRS-2 are the most extensively studied and are known to have overlapping but also distinct biological roles. These proteins act as essential mediators for both insulin and IGF-1 receptor signaling (55).

While they share common downstream signaling pathways, insulin, and IGF-1 receptors have distinct physiological roles. Both pathways are implicated in diseases like diabetes, cancer, and growth disorders. Shc and IRSs not only facilitate the retention of activated IGF-1R at the cell membrane but also initiate downstream signaling cascades associated with the IGF axis (53). These adaptor proteins become phosphorylated and activated facilitating the recruitment of Grb2/SOS (son of sevenless, the guanine nucleotide exchange factor) complex which activates Ras by promoting the exchange of GDP for GTP on this proto-oncogene, converting it to its active form. In this configuration, Ras-GTP binds to and activates the serine/threonine kinase RAF, which in turn will activate MEK (MAPK/ERK kinase), and consequently, this kinase phosphorylates and activates MAPK/ERK (extracellular signal-regulated kinase) (56). Activated MAPK translocates to the nucleus, where it phosphorylates and activates transcription factors such as Elk-1, c-Myc, and c-Fos. This activation leads to the transcription of genes involved in cell cycle progression, such as cyclin D1, and genes that promote proliferation, including those encoding ribosomal proteins and metabolic enzymes (57). Additionally, MAPK phosphorylates proteins involved in the regulation of the cell cycle, including cyclins and cyclin-dependent kinases, promoting in this way progression through the cell cycle phases. MAPK activation can also phosphorylate and inhibit pro-apoptotic proteins, thereby promoting cell survival and proliferation (58). Overall, MAPK-mediated phosphorylation of these targets enhances cellular proliferation by stimulating gene expression, promoting cell cycle progression, and supporting cellular growth and survival pathways.

However, the association of these two canonical pathways to specific outcomes is largely an oversimplification, as they are recruited for a wide variety of functions involving numerous other targets that are constantly updated (Table 1). Among these, PKC is probably the most commonly cited. Additionally, these two branches of IGF-1-R canonical pathways show cross-talk under specific circumstances (59). While the tyrosine kinase activity of IGF-1R is needed for downstream AKT activation, MAPK activation takes place without it (60), requiring in this case the C-terminal region of the receptor (61).

**Table 1:** Non-canonical downstream targets of IGF-1R activated by PI3K/AKT or Ras/MAPK pathways

Target	Pathway	References
AMPK	AKT	(507)
EZH (methyltransfe-rase)	AKT	(508)
FoxG1 (transcription factor)	AKT	(509)
PKC	AKT/MAPK	(510, 511, 512, 513, 514, 515, 516, 517)
PKA	AKT	(510)
PLCγ/CREB	AKT	(511, 518)
c-JUN kinase	AKT/MAPK	(519)
MEF2C (transcription factor)	AKT	(520)
HDAC5 (deacetylase)	AKT	(521)
P63 (transcription factor)	AKT	(522)

As with any other signaling cascade, feedback regulation of IGF-1R activity is regulated by differential phosphorylation of IRS-1 at serine residues mediated by different kinases, which results in functional uncoupling and desensitization of IGF-1R (62). Alternative desensitization pathways include proteasome-mediated degradation of IRS-2 (63), direct dephosphorylation of IGF-1R by SHP-2 (64), its phosphorylation at serine residues by GSK-3β (65), or its inactivation by changes in the ganglioside composition of the cell membrane (66). When analyzing the biological significance of these pathways, it is often assumed that experimentally altering the activity of key components, such as IRSs—shared by other extracellular signals (67, 68, 69, 70) and exhibiting IGF-1-independent roles (71)—is equivalent to modifying ILP signaling. This common oversimplification is an important confounding factor to consider. Finally, IGF-1R signaling may be modulated by processes such as acetylation (72), glycosylation (73), prenylation (74), neddylation (75), SUMOylation (76) or through interactions with a specific cell membrane inhibitor named inceptor (77). These alternative modulatory routes of IGF-1R activity require further study.

## Non-canonical IGF-1R signaling

We use the term “non-canonical signaling” to describe signal transduction pathways of IGF-1R that operate either independently or upstream of PI3K/AKT—Ras/MAPK routes. We will also include *pseudo-*canonical signaling; *i.e.*: non-canonical pathways where canonical signaling routes have not been entirely ruled out (Fig. 1*B*).

The first non-canonical pathway characterized decades ago includes heterotrimeric G-proteins (27, 78), widely confirmed in different biological contexts and cell types (79, 80, 81, 82) and even in specialized cell structures (81). This pathway includes the modulatory protein β-arrestin involved in IGF-1R internalization (83), and the adaptor protein RACK 1 (84). Based on these observations, IGF-1R activity was proposed to be a functional tyrosine kinase/G protein-coupled receptor (GPCR) with biased signaling, as shown in classical GPCR activities (85). Closely intertwined with this type of modulation of IGF-IR activity is its transactivation in response to agonists of G protein-coupled receptors. IGF-1R transactivation can be done by several GPCRs, including thrombin (86), bombesin (87), angiotensin II (88), or GABA_B_ receptor (89). Upon ligand binding, GPCRs activate heterotrimeric G-proteins that initiate a cascade of intracellular signaling events, ultimately leading to the transactivation of IGF-1R. These pathways include direct phosphorylation of IGF-1R by tyrosine kinases such as Src (90), generation of second messengers (91), metalloprotease-mediated tumor cell invasion (92), and scaffolding by β-arrestins (93), all of which contribute to the activation of IGF-1R and its downstream signaling pathways.

Other pathways apparently independent of canonical IGF-1R signaling include the phosphatase calcineurin and the transcription factor NFATc1, involved in the remodeling of skeletal muscle (94), or the phosphatase PTPα, instrumental in cell movement (84). In addition, IGF-1R shows wide cross-talk activity with tyrosine kinase receptors (95, 96), non-receptor tyrosine kinases such as the Src protein network (97), and other receptors such as for GABA, P2Y12 (98), hepatocyte growth factor (99), estrogen (100), angiotensin II (101), pituitary thyrotropin stimulating hormone (TSH) (102), neuropeptide Y (103), androgen (104) or lysophosphatidic acid (LPA) (105). Considering that extracellular signals, such as neurotensin, can transactivate IGF-1R in colonic epithelial cells to phosphorylate AKT and activate NFκB (106), and that intracellular pathways, including PTEN-induced kinase 1 (107) and leukemia-associated Rho guanine nucleotide exchange factor (108)—to name a few, may also interact with IGF-1R, the complexity of the IGF-1R pathway is dismayingly ample.

Pseudo-canonical pathways include the serine-threonine kinases PKC, which, for example, intervenes in the modulation of Ca^2+^ L-channels by IGF-1 (109), and PKA, which links G protein-coupled signaling with IGF-1R signaling (110), and mediates modulation of organic anion transporter three in the kidney (111). Other pathways include the phosphatase calcineurin, which is downstream of IGF-1R activation in mesangial renal cells (112) and astrocytes (113), or the multi-cargo membrane protein transporter LRP1, which participates in glucose handling by IGF-1R signaling in astrocytes and Muller glial cells (114, 115), and in adipogenesis (116). Furthermore, activation of IGF-1R results in its ubiquitination (117) and internalization that may lead to its recycling, degradation, or translocation to different cellular compartments (118, 119, 120). All these steps include interactions with different proteins (121) *via* endocytosis by clathrin-coated pits (122), in a cell- and context-dependent manner (120).

We also refer in this section to those IGF-1R pathways located at specific cell organelles rather than in the cell membrane/cytoplasm. Indeed, different constituents of the IGF-1R signaling machinery have been found in the mitochondria (123), the cell nucleus (124), and the Golgi apparatus (125). Although the functional significance of IGF-1R signaling outside the cell membrane is in general little understood, we herein describe the scattered and scarce available information on this alternative signaling pathways.

### Nuclear signaling

The observation that activation of IGF-1R induces its translocation to the cell nucleus in a SUMOylation-dependent process that is independent of its kinase activity (126) opened new downstream pathways that are slowly being unveiled. Beyond their apparent involvement in cancer pathology, the anti-inflammatory activity of IGF-1 in mouse models of Alzheimer's disease (127), or the proliferation of intestinal cells (128) depends also on IGF-1R nuclear signaling. The nuclear presence of IGF-1R – IR hybrids has also been documented and appears to be modulated by insulin (129). MAPK and IRSs canonical pathways seem to participate in this process, as their presence has been described in the cell nucleus (130). Collectively, the data point to a transcriptional modulatory role of IGF-1R in the cell nucleus (28, 131). Indeed, nuclear IGF-1R binds directly to its promoter to upregulate its expression (132).

### Mitochondrial signaling

While this organelle is a classical target of canonical signaling by IGF-1, including early observations that IGF-1 induces the translocation of Raf to the mitochondria (133, 134), their role in physiological IGF-1R activity has been reinforced by subsequent observations of the presence of IGF-1R itself (123, 135, 136), and its downstream targets such as AKT and GSK-3β in mitochondria (137).

### IGF-1R in the Golgi

The presence in the Golgi of an ILP receptor distinct from the insulin receptor was reported decades ago (138). This organelle is involved in intracellular trafficking and sorting of IGF-1R to the cell membrane and the cell nucleus (128), and is important in the oncogenic activity of IGF-1 (see below). However, IGF-1R signaling also takes place at the Golgi and is involved in cell migration (125).

## Ligand-independent actions of IGF-1R

While early observations hinted to a role of IGF-1R on its own in muscle differentiation (139), the first report that specifically addressed ligand-independent actions of IGF-1R (and IR) describing their pro-apoptotic activity, went mostly unnoticed, even though it originated from a prominent group in ILPs signaling (140). Subsequent work showed its role in gene imprinting (141), while more recently, our group unveiled both ligand-dependent and independent actions of IR and IGF-1R in relation to glucose uptake by the brain (96, 115). This dependence signaling (140) includes combined activation of IR and IGF-1R that recruits MAPK and Protein Kinase D and a set of complex protein-protein interactions involving the small GTPase Rac1, L-RP1, and Glucose transporter 1 (GluT1). This pathway remains incompletely characterized, but other authors confirmed that LRP1 and GluT1 participate in IGF-1 receptor signaling in glucose uptake by another type of astrocyte, the Muller glial cell in the retina (114), and that IGF-1R is also involved in glucose metabolism in skeletal muscle (142). Building on these observations, we recently proposed that the ambivalent role of IGF-1R as a ligand-dependent and independent receptor may help explain apparently contradictory observations regarding its actions in health and disease (143).

## IGF-1 signaling along evolution

**Figure 2:**
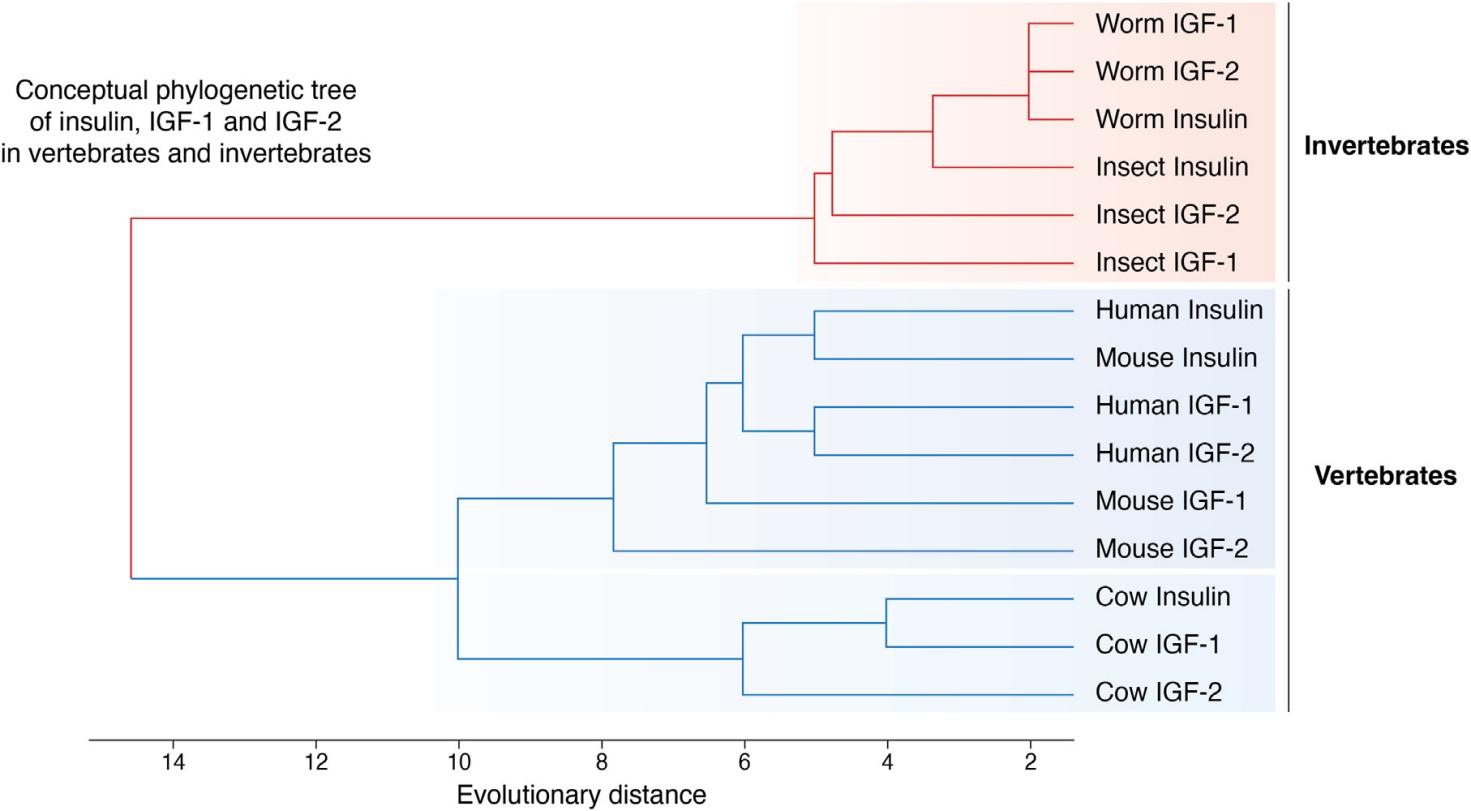
**Conceptual phylogenetic tree of ILPs.** Evolutionary relationships across vertebrates and invertebrates show a clear distinction between them. Phylogenetic branches in invertebrates are shorter and group more closely, suggesting less divergence compared to the vertebrates. The evolutionary distance between insulin, IGF-1 and IGF-2 is indicated by higher branches showing greater divergence, meaning that peptides with branches that join closer to the left of the tree are more evolutionary distant from each other. Among mammals, three species were compared to illustrate that humans and mice are more closely related than the cow, which diverged earlier in evolutionary time. The graph was generated using ChatGPT-4 after extracting evolutionary data of ILPs from UniProt. Raw data was run through MATLAB software. Note that the use of the terms IGF-1 and IGF-2 in worms and insects is merely conceptual as- in invertebrates ILPs are identified following species-specific nomenclature.

**Figure 3:**
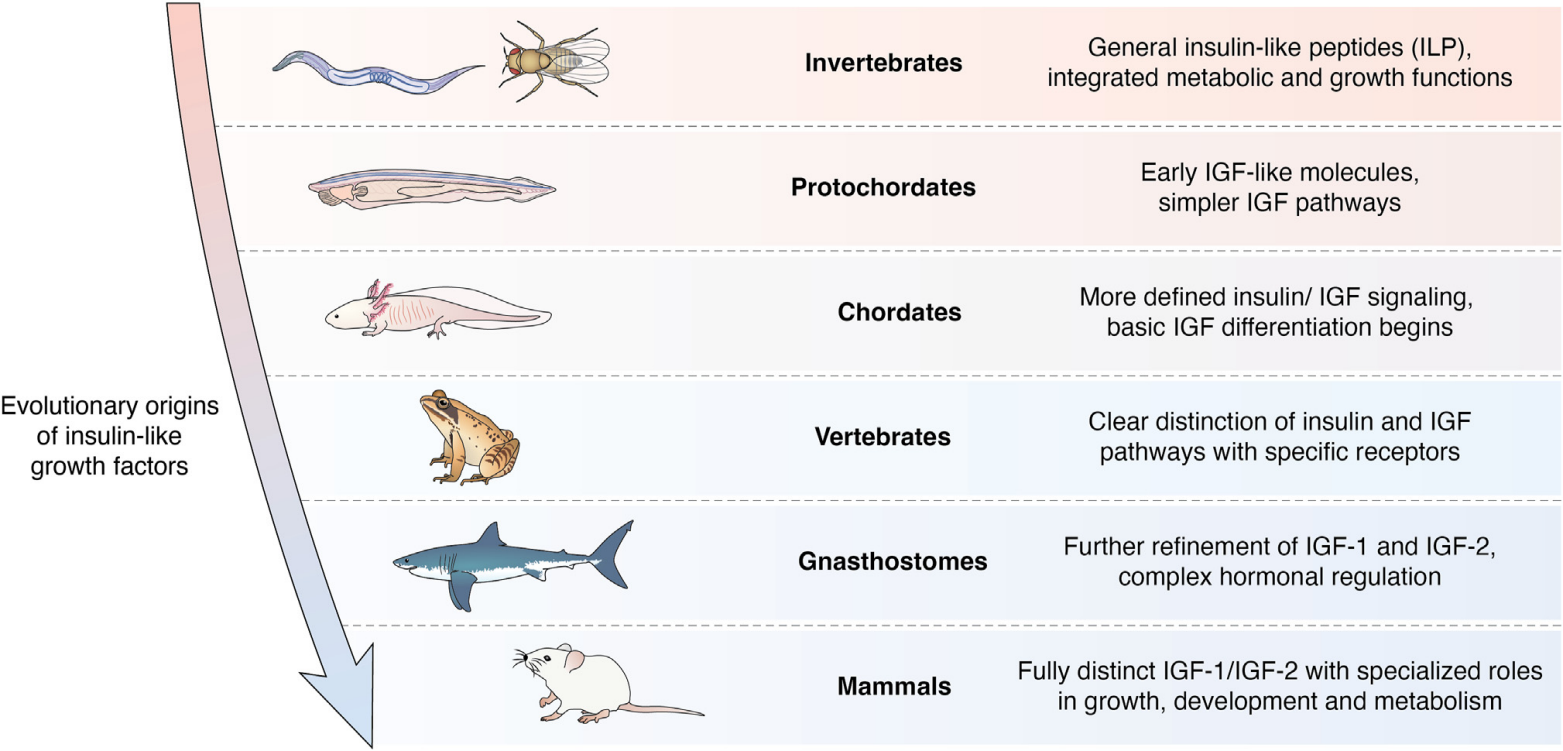
**Insulin peptides along evolution.** ILPs are already found in *non-chordata* (invertebrate) phyla such as *arthropoda* (insects) and *annelida* (worms) where they present an organization consisting of more ligands than receptors. However, most of the available information is within the phylum *chordata* including protochordates and vertebrates. In turn, detailed evolutionary information is more available in the latter where complex signaling pathways, novel receptors, and less ligands have been documented. Representative acquisitions along with evolution are included.

Signaling by ILPs is so well conserved along evolution (Fig. 2), that human insulin can activate daf-2, the worm ILP receptor (144), and viruses have hijacked IGF-IR (4). Evolution has transformed an ILP system based on more ligands than receptors into a one ligand/one receptor network (Fig. 3). Thus, invertebrates such as *D. melanogaster* or *C. elegans* have from 8 to 40 ligands (145, 146) and a single ILP receptor, respectively. Many other invertebrate species show also more ligands than receptors (147), while some others developed receptor duplications, as seen in vertebrates, with one receptor for insulin and another for IGF-1 with high structural homology and shared intracellular signaling (148, 149). In specific subgroups such as eusocial insects, differential expression of ILP ligands takes place among castes (150). In vertebrates, each ligand has its own receptor, despite a certain degree of cross-reactivity and the existence of insulin-IGF-1 hybrid receptors. This evolutionary trait remains largely unexplained, although the most parsimonious explanation is that this arrangement provides evolutionary advantages. However, while the organizational advantages of invertebrate ILPs networks have been explained (151), no specific mechanisms underlying the purported advantages of ligand/receptor pairs in vertebrate ILPs have been addressed yet. At any rate, downstream molecules in canonical PI3K/AKT signaling are highly conserved, with invertebrates showing homologs for all those found in vertebrates, including the docking IRSs proteins, the kinases PI3K and AKT, and the transcription factor Foxo (152).

Despite its large number, each worm ILP may fulfill varied roles (153), which reflects the huge variety of actions that ILPs play even in a simple organism. In higher invertebrates such as *D. melanogaster*, ILPs fulfill new roles such as state-dependent modulation of sensorimotor processing (154). Modulation of brain states through IGF-1 signaling has recently been proposed by us to be also conserved in mammals (155). Furthermore, *Drosophila* insulin peptides (dILPs) are involved in anti-microbial responses (156), a role also found in their vertebrate counterparts (157). Taken together, these findings suggest that ILPs fulfill conserved roles even in evolutionarily advanced functions. In vertebrates, the multitasking of ILPs is maintained through ILP ligand/receptor pairs using a plethora of intracellular signaling pathways, or in specific cases *via* gene duplication of IGF-1R, as observed in fishes (117).

## Context-dependent activity of IGF-1R

Responses to extracellular modulators are context-dependent. For IGF-1R, this characteristic is particularly varied, given the wide spectrum of biological actions of its main ligand, IGF-1. Among the different possible contexts, we will delve in the two best studied: cell types and health/disease. In addition, IGF-1R activity is often found to be sexually dimorphic (158, 159, 160), but this trait remains largely understudied, with scarce information available, making it difficult to provide a meaningful overview. The fact that estrogen receptors interact with IGF-1R activity (100) probably helps understand underlying mechanisms. Also, as IGF-2 is paternally imprinted in a tissue-specific manner (40), and can also signal through the IGF-1R, this may contribute to sex differences in IGF-1R activity. However, much more work is needed to clarify the molecular basis of sexually dimorphic IGF-IR activity.

Ontogeny is also a key contextual aspect in IGF-1 actions, as illustrated by its differential actions along life stages (161, 162). Indeed, it is well known that IGF-1R mediates developmental actions such as embryogenesis, cell proliferation and differentiation, and organogenesis (163). In the adults, IGF-1R activity can be generally described as homeostatic and cytoprotective, significantly contributing to the functional integrity of the mature organism. Along with this role, it has recently been shown in invertebrates that IGF-1R activity is modulated by external factors such as temperature and diet (162), and in mammals, by glucose levels (164). During aging, its role has been extensively studied (165, 166), although it still stirs considerable controversy (167). We recently proposed that IGF-1R activity in aging may be partially detrimental due to a gradual predominance of its ligand-independent actions over its ligand-dependent actions as a result of the age-associated resistance to IGF-1 and insulin (143). However, the role of IGF-1 and its receptor in aging and associated diseases is beyond the scope of this review as it is a major chapter in IGF-1R biology, extensively discussed elsewhere.

### IGF-1R activity in non-tissue-specific cells

**Figure 4:**
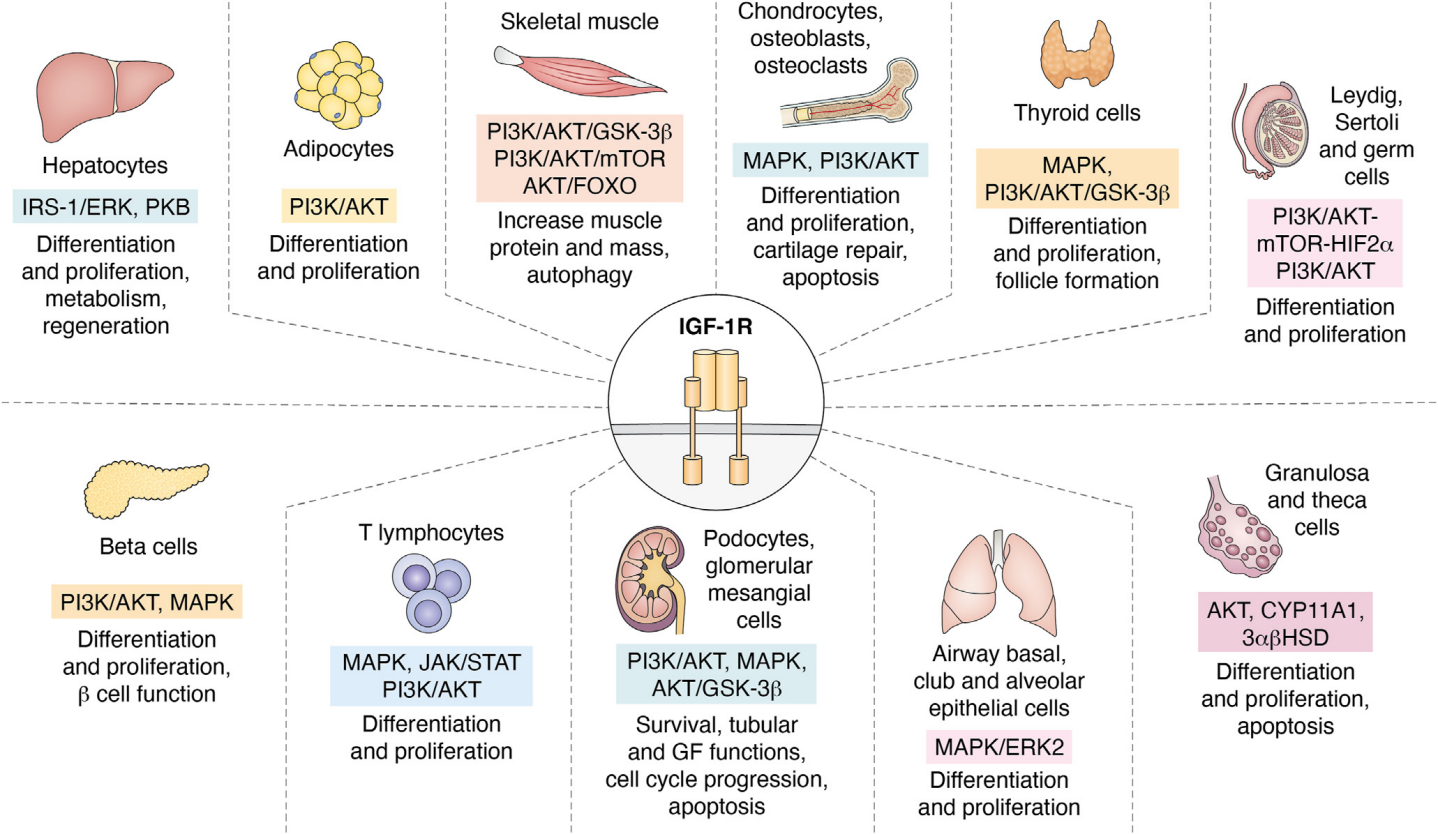
**Main IGF-1R signaling pathways in tissue-specific cells.** In adipocytes, IGF-1R signaling through PI3K/AKT promotes the differentiation of preadipocytes into mature adipocytes. In hepatocytes, IGF-1R engages the ERK pathway *via* the recruitment of Grb2 and SOS, leading to the activation of Ras, Raf, MEK, and ERK. Activated ERK translocates to the nucleus and upregulates cyclins D1 and A, driving cell cycle progression and hepatocyte proliferation. In myocytes, IGF-1R signaling through AKT promotes cell survival through mTOR which also enhances protein synthesis and prevents apoptosis, supporting muscle growth and maintenance. Additionally, AKT inhibits GSK-3β further promoting cell survival and preventing muscle atrophy. In chondrocytes, osteoclasts, and osteoblasts, IGF-1R signaling through PI3K/AKT and MAPK promotes differentiation and proliferation, cartilage repair, and prevents apoptosis. IGF-1R enhances thyroid cell survival, growth, proliferation, and follicle formation through MAPK and PI3K/AKT/GSK3β. In testes, IGF-1R signaling through the PI3K/AKT/mTOR pathway promotes survival, growth, and proliferation of Leydig, Sertoli, and germ cells. In the ovaries, IGF-1R promotes growth, and proliferation through AKT and stimulates the expression of steroidogenic enzymes CYP11A1 and 3βHSD in granulosa and theca cells. IGF-1R modulates the development and differentiation of various lung cell types, including airway basal cells, club cells, alveolar epithelial cells, and fibroblasts through MAPK/ERK2. In the kidney, IGF-1R promotes podocyte cell survival and integrity of the glomerular filtrating barrier through PI3K/AKT and MAPK. IGF-1R is involved in the development of T-lymphocytes within the thymus, as well as in the proliferation of mature T cells through MAPK, JAK/STAT, and PI3K/AKT. Finally, IGF-1R promotes the differentiation and proliferation of β-cells and regulates their function through PI3K/AKT and MAPK pathways.

Detailed information on cell-specific expression of human IGF-1R is available at https://www.proteinatlas.org/ENSG00000140443-IGF-1R/tissue, while for other species available data is less comprehensive. As stated above, most cell types, if not all, express this receptor. Albeit cell-specific roles of IGF-1R and underlying pathways are not usually emphasized in the literature, analysis of available evidence indicates that IGF-1R activity is exquisitely cell-dependent (Fig. 4). An important drawback of most of these studies is that data have been obtained using *in vitro* approaches, with *in vivo* significance still pending to be confirmed.

We will first describe IGF-1R routes that have been characterized in cell types that are present in multiple tissues and organs such as macrophages, endothelial cells, epithelial cells, and fibroblasts, under non-pathological conditions. Those under pathological conditions, mostly in relation to cancer, will be described in the corresponding section (see below).

As a rule, the role of IGF-1R in these four types of cells is strikingly broad and many of the pathways involved remain to be described in greater detail. We will provide various examples to illustrate the diversity of routes employed by IGF-IR.

#### Macrophages

These monocyte-derived myeloid cells of the immune system colonize tissues such as skin, lung, spleen…etc. Monocytes migrate to tissues in response to inflammation or infection and differentiate into macrophages, while tissue-resident macrophages develop from precursor cells during embryonic development and are seeded into various tissues, where they persist throughout life. The PI3K/AKT pathway is essential for the survival and proliferation of macrophages, helping them to persist in the tissue environment and perform their functions effectively (168). Activated AKT in macrophages phosphorylates and inactivates several pro-apoptotic proteins, such as Bad and caspase-9 (51). Phosphorylation of Bad prevents its binding to Bcl-2 and Bcl-xL, promoting cell survival. AKT enhances the transcription and translation of survival genes and proteins such as mTOR (50), supporting cell growth and proliferation. It also inhibits GSK-3β, preventing the degradation of pro-survival transcription factors like β-catenin (51). The IGF-1R/PI3K/AKT pathway is also involved in macrophage activation (169).

#### Endothelial cells

These cells line the interior surface of blood and lymphatic vessels, forming a barrier between the blood or lymph and the surrounding tissues. IGF-1R is expressed by endothelial cells in both large and small vessels, and its levels are higher than those of IR, although it has been reported that insulin acts through IGF-1R to activate the MAPK/p38MAPK route and promote endothelial growth (170). Most actions of IGFs in these cells are mediated by IGF-1R, though some actions of IGF-2 are mediated by the IGF-2/mannose 6-phosphate receptor. IGFs activate several signaling pathways, including MAPK, PI3K/AKT, NO, and PKC, leading to endothelial cell migration, survival, nutrient uptake, and tube formation (171). Thus, IGF-1R acts through the PI3K/AKT pathway to promote angiogenesis (172), or to decrease apoptosis and microvascular damage (173). The PI3K/AKT pathway is indeed a crucial mediator of the angiogenic phenotype. AKT signaling is essential for the normal transition from the G1 to S phase of the cell cycle and proliferation in endothelial cells (174), while *via* PI3K/Pak1/MAPK, IGF-1R promotes the transcription factor RUNX_2_ involved in the migration of these cells (175). The MAPK pathway is also involved in the stabilization of nascent vessels by IGF-1 (176). In retinal endothelium, IGF-1R acts through PI3K/AKT/GSK-3β/CREB to promote cell progression and extracellular matrix molecules (177), whereas endothelial nitric oxide synthase (eNOS) is activated in human endothelial cells through PI3K (178) and caveolin-1 (179), and is likely involved in blood pressure regulation (180).

IGF-1 enhances glucose uptake, stimulates DNA synthesis, facilitates cell migration, promotes inflammatory and vasodilatory responses, and induces angiogenesis in vascular endothelial cells (181, 182). Evidence suggests that IGF-1 functions as a vasodilator *in vivo*. In patients with low IGF-1 levels, impaired flow-mediated arterial dilation was observed, which is dependent on the endothelium and mediated by nitric oxide (NO) (183). Further, circulating IGF-1 levels in humans are considered valuable biomarkers for the development of cardiovascular diseases. For instance, low serum IGF-1 is linked to hypertension and early cardiovascular complications in female patients with rheumatoid arthritis (184), whereas elevated IGF-1 plasma levels are correlated with a reduced risk for hypertension in non-diabetic women patients (185). Mice with decreased circulating IGF-1 levels also exhibited higher blood pressure compared to control mice. This increase in blood pressure was attributed to impaired endothelium-dependent, NO-mediated vasorelaxation and increased expression of the vasoconstrictor endothelin (186). *In vitro*, IGF-1 stimulated NO production *via* PI3K activation and eNOS in human umbilical vein endothelial cells (178). In short, this growth factor contributes to blood pressure regulation (180, 187).

Senescence is a cellular state of growth arrest and unresponsiveness to growth stimuli while remaining metabolically active (171). IGF-1 inhibits oxidative stress-induced senescence of human aortic endothelial cells (188). IGF-1 reduces senescence through the FAK (focal adhesion kinase)/MAPK axis and enhances eNOS and vascular endothelial growth factor (VEGF) production in endothelial progenitor cells (189, 190). Specifically, IGF-1 production is increased *via* activation of the FAK/MAPK pathway, which occurs following a reduction in calcium influx (191). This suggests that IGF-1 plays a crucial role in rescuing the angiogenic potential of senescent endothelial cells, promoting their therapeutic efficacy in angiogenesis. As with all IGF-1R actions, its diverse effects on endothelial cells play a crucial role in tissue repair, such as in myocardial ischemia. However, these effects can be harmful in malignancies, where IGF-1R may promote tumor growth, or in diabetic retinopathy (192).

#### Epithelial cells

These cells are present in the majority of tissues and exert a striking variety of actions such as cytoprotection, secretion, or transport, to name a few. The role of IGF-1R in these cells is multifaceted and significant for various cellular processes. In epithelial cells of the intestine, IGF-1R recruits Ras and PI3K, which are involved in gene transcription, cell proliferation, and apoptosis (193). Thus, IGF-1 is essential for restoring the integrity of the intestinal epithelium (194). In these cells, IGF-1R undergo-clathrin-mediated endocytosis and then entere Rab-5-positive endosomes. IGF-1 and/or IGF-1R entere the cell nuclei through the nuclear pore complex and promote cell proliferation (128). Furthermore, IGF-1 and the intestinal epithelial IGF-1R are required for glucagon-like peptide-2 (GLP-2)- induced increases in intestinal growth and proliferation, and its stimulatory effects on barrier function and microvillus length (195). Furthermore, IGF-1R plays a significant role in enterocyte lipid handling, under basal conditions and in response to GLP-2 and Western diet-feeding (196).

In alveolar epithelial cells of the lung, IGF-1R modulates endocytosis through the PI3K/AKT pathway (197), and participates in their senescence (198). These cells are important for the gas exchange role of the lungs, production of pulmonary surfactant, fluid balance, and immune defense. In the epithelium of the embryonic lens, IGF-1R is prominently expressed in the region where the initiation of differentiation occurs (199) and it plays a role in signaling the process of lens differentiation (200). Thus, activation of the IGF-1R/NFκB pathway maintains caspase-3 at low levels to induce the initiation of lens epithelial cell differentiation (201). IGF-1 and insulin trigger also the PI3K pathway in lens epithelial cells (202) where is actively involved in lens epithelial cells survival and proliferation (203).These epithelial cells are crucial for lens homeostasis and transparency.

#### Fibroblasts

These cells provide structural support to tissues, produce extracellular matrix (ECM) components, help transmit mechanical forces and participate in angiogenesis, repair and inflammation. A substantial part of the information of the role of IGF-1R in fibroblasts has been gathered in embryonic stages, where its ligand is a mitogenic signal. IGF-1R acts through PI3K/AKT to stimulate the GRP78 chaperone (204) or the prolyl isomerase Pin1 (205). In adult cells, IGF-1R acts through various pathways. Thus, in skin fibroblasts and tenocytes IGF-1R acts through canonical MAPK and PI3K pathways as a mitogenic signal (206). In lung fibroblasts, IGF-1R promotes cell proliferation activating c-Fos and the early growth response (EGRs) transcription factors (207), whereas in corneal fibroblasts stimulates N-cadherin (208) through undescribed pathways. In intestinal fibroblasts, IGF-1R stimulates collagen one synthesis through MAPK (209).

### IGF-1R activity in tissue-specific cells

In accordance with its ontogenetic-dependent action, IGF-1R mediates both developmental and homeostatic/cytoprotective roles of its ligand IGF-1 in all tissues. We now describe tissues and pathways involved where evidence is available (Fig. 4), but we predict that IGF-1R activity will be eventually defined as a universal regulator in all tissues.

#### Fat

Insulin and IGF-1 are crucial for the development and differentiation of white and brown adipose tissue (WAT and BAT) (210). IGF-1 stimulates both cell growth and lipogenesis during the differentiation of human mesenchymal stem cells into adipocytes *in vitro* (211). In preadipocytes, the expression of IGF-1R is higher than that of IR, but in mature adipocytes, this pattern is reversed (25, 212). IGF-1R signaling is crucial for initiating adipocyte differentiation through AKT (213). In addition, mice with a fat-specific deletion of the IR show a reduction in both WAT and BAT mass, while mice with a fat-specific deletion of IGF-1R exhibit a slight increase in adipose tissue mass and overall body growth (214). In turn, deletion of both receptors in fat leads to a significant reduction in the mass of both WAT and BAT and resistance to obesity, even when subjected to a high-fat diet (215). However, a recent study highlighted that inhibition of IGF-1R during a high-fat diet (HFD) can lead to a lipodystrophic phenotype characterized by a failure in WAT lipid storage (216) while mice lacking only IR or both IR and IGF-1R exhibit a lipodystrophic phenotype, characterized by severe diabetes, insulin resistance, and abnormal fat distribution in muscle and liver (217, 218).

#### Liver

While the liver appears to be the major producer of IGF-1, its receptor is strongly expressed only during development and is much more weakly expressed in adults, being virtually undetectable in adult hepatocytes (219). In contrast, IGF-1 is abundantly produced by mature hepatocytes in adults (220), where its secretion is regulated through the energy-sensing LKB1–AMPKα1 pathway and consequent activation of the IGF-1R/AKT pathway (221). In mice with liver-specific inactivation of the *Igf1* gene, the absence of the acid-labile subunit (ALS) delays hepatic regeneration (222). Additionally, elevated levels of IGFBP-1 have been observed during liver regeneration in mice (223), and IGFBP-1 null mutants exhibit abnormal liver regeneration (224, 225). These findings suggest that changes in the bioavailability of IGF-1 are crucial for the proliferation of liver cells. Thus, IGF-1R controls the proliferation, differentiation, and cell survival of hepatocytes. IRS-1/MAPK signaling is the intracellular pathway controlling the cell cycle *via* cyclin D1 and cyclin A (226), whereas the IGF-1R/PI3K/AKT pathway is activated by both IGF-1 and IGF-2 to promote hepatocyte differentiation from their precursors (227). IGF-1 can also indirectly regulate hepatocyte metabolism through the modulation of GH inputs (228). This pituitary hormone interacts with various target tissues, including hepatocytes, to stimulate IGF-1 synthesis. GH regulates the autocrine secretion of IGF-1 from hepatocytes through a GH receptor/JAK2/PLC signaling pathway (229). It is important to note that pituitary somatotropes express both IGF-1R and IR (33). Mice with somatotrope-specific knockout of IGF-1R (32, 33) showed elevated levels of pituitary GH and GHRH receptor mRNA, and this was associated with an increase in circulating GH and total IGF-1 levels. These studies demonstrate that IGF-1 directly inhibits pituitary production of GH.

#### Skeletal muscle

Skeletal muscle cells, also known as myofibers, are postmitotic, meaning they do not undergo cell division. Their size is determined by the balance between protein synthesis and degradation. IGF-1R activity regulates muscle size and plays a critical role in muscle function, contributing to many of the benefits of physical activity (230). In myocytes, IGF-1R promotes cell survival *via* the PI3K/AKT pathway (231) and enhances skeletal muscle protein synthesis through the PI3K/AKT/mTOR and PI3K/AKT/GSK-3β pathways (232). These pathways are downregulated in various muscle atrophy conditions (233, 234). Interacting with this pathway is myostatin, a member of the transforming growth factor-β superfamily, that is primarily secreted by skeletal muscle and negatively regulates muscle mass (235). Myostatin inhibits IGF-1R-dependent AKT phosphorylation, resulting in decreased protein synthesis and reduced cell size (236), whereas the hypertrophic effect of IGF-1 is greater in the myostatin null background (237). While skeletal muscle-specific IGF-1R null mice showed no altered body weight or muscle mass, the double knockout of IGF-1R and IR showed a marked decrease in skeletal muscle mass and fiber size and died earlier, likely due to respiratory failure (142).

#### Bone

This tissue is a main target of IGF-1, affecting the activity of its major cell types (238). Chondrocytes in the long bones express IGF-1R and its major ligand, IGF-1. Global deletion of IGF-1 and its receptor results in skeletal defects and cartilage changes, including reduced bone length (239). Further, chondrocyte-specific IGF-1 knockout mice showed changes in the longitudinal growth and width of bone as well as a decrease in bone mineral density (240). Cartilage-specific IGF-1R knockout mice died shortly after birth and showed disorganized chondrocyte columns, delayed ossification, and vascular invasion, decreased cell proliferation, increased apoptosis, and increased expression of parathyroid hormone-related protein in their growth plate (241), in an IGFBP-regulated fashion (242). Using a tamoxifen-inducible model to delete IGF-1R in chondrocytes 1 week postnatally revealed a significant reduction in growth plate chondrocyte proliferation and differentiation (243). IGF-1R also can inhibit NFκB activity in these cells *via* regulation of the MAPK and PI3K/AKT signaling pathways and prevent apoptosis by suppressing ROS production (244). During osteoarthritis, chondrocyte mechano-sensitivity is greatly altered, which can contribute to cartilage degradation. Modulation of the ion channel transient receptor potential vanilloid four by IGF-1 might help in restoring or maintaining normal mechano-transduction pathways (245). IGF-1 can significantly enhance chondrocyte division and proliferation from various sources and stimulate the synthesis of type II collagen and aggrecan in chondrocytes (246).

Enhancing IGF-1R expression through modulation of various pathways could be a potential therapeutic strategy to promote cartilage repair and counteract the progression of osteoarthritis. The regulation of IGF-1R by circRNAs such as hsa_circ_0045714 and microRNAs like miR-193b highlights a complex interplay of molecular mechanisms that control chondrocyte activity and cartilage integrity (247). MiR-26a can suppress the proliferation of chondrocytes and promote their apoptosis by regulating IGF-1 (248). Genetic and pharmacological activation of nuclear-localized sirtuin six promotes pro-survival and pro-anabolic IGF-1/AKT activation in human chondrocytes (249). Intra-articular administration of high-dose of IGF-1 contributed to a significant increase in mandibular growth and condylar bone quality and quantity in growing rats (250). Overall, IGF-1 signaling pathways regulate chondrocyte proliferation and differentiation, synthesis of cartilage matrix, and reduction of apoptosis.

In osteoblasts, a specialized cell that plays a crucial role in bone remodeling and resorption, IGF-1R plays a role through the PI3K/AKT pathway in differentiation and proliferation (251), affecting various mechanisms such as stimulation of their precursors, and enhancement of osteogenic markers such as alkaline phosphatase, osteocalcin, and collagen type I. IGF-1R prevents senile osteoporosis by promoting the proliferation of senescent osteoblasts *via* PI3K/AKT (252) and controls bone mass (253) and mineralization (254). Its ligand IGF-1 plays a significant role in the differentiation and proliferation of osteoblasts, through various mechanisms including stimulation of osteoblast precursors, and enhancement of osteogenic markers. IGF-1 stimulates cell proliferation and differentiation/extracellular matrix mineralization through the MAPK and PI3K/AKT pathways (255). In addition, IGF-1 enhances osteoclast formation, bone resorption, and remodeling by increasing the synthesis of receptor activators of NFκB ligand by these cells (256). IGF-1 signaling is crucial for sustaining the proliferation of both chondrocytes and osteoblasts, ensuring proper endochondral ossification (257). Conditional IGF-1 knockout mice, where IGF-1 is deleted in osteoblasts, demonstrated a decrease in bone formation, resulting in an overall reduction in bone mass (258). Conversely, overexpression of IGF-1 driven by the Col1α1 promoter led to an increase in the length of long bones and cortical width, with minimal impact on trabecular bone. Additionally, calvarial bone width was increased, indicating that overexpression of IGF-1 in osteoblast lineage cells promotes bone growth (259). Further, in cooperation with IGFBP-2, IGF-1 stimulates osteoblast differentiation through AMPK activation (260). IGF-1 has also been shown to enhance the expression of osteogenic markers, including alkaline phosphatase, osteocalcin, and collagen type I. These markers are characteristic of mature osteoblasts and are involved in the formation of bone matrix. In addition, IGF-1 significantly amplified Bone Morphogenetic protein-induced osteogenic differentiation in murine pre-osteoblasts (261). Notably, the decline in mitogenic activity and osteo-blastogenic potential of bone marrow mesenchymal stem cells associated with aging was restored by administering higher doses of IGF-1 (262).

Another bone cell targeted by IGF-1R is osteoclasts. These specialized cells are responsible for breaking down bone tissue, a process essential for the maintenance, repair, and remodeling of bones. Numerous studies have demonstrated that IGF-1 and IGF-1R are expressed in osteoclasts, and that IGF-1R plays a role in osteoclast differentiation (263). Knockout of IGF-1 specifically in osteocytes affected bone longitudinal and cortical growth along with a decrease in the calvarial bone growth rate (264).

#### Thyroid gland

This endocrine gland is considered a classical target of IGF-1. This growth factor is involved in its development and maintenance, acting in concert with TSH (102), and other hormones (see below), through IGF-1R canonical signaling involving IRS-2 (265), and downstream targets such as NFκB (266). Although most work has been conducted *in vitro* (*i.e.*: FRTL5 cells) and involves insulin, IGF-1R appears to recruit also MAPK to impact thyroid gland metabolism (267). In a study with conditional thyroid-specific IGF-IR and IR double knockout mice, neonatal thyroid glands were observed to be smaller, exhibited reduced expression of the thyroid-specific transcription factor FOXE1, and showed defective folliculogenesis (268). Thus, both IGF-IR and IR are essential for proper follicle formation in the developing thyroid. Indeed, IGF-I stimulates DNA synthesis and thyroid cell proliferation, working synergistically with epidermal growth factor (269). Additionally, IGF-I increases the levels of thyroid transcription factor-2, similar to the effects of insulin (267, 270).

#### Testes

The IGF system seems to influence testicular development and function acting in concert with the pituitary follicle-stimulating hormone (FSH) (271). IGF1-R plays a role in Leydig, Sertoli, and germ cell proliferation and differentiation (272, 273, 274, 275, 276). Additionally, Leydig cells can secrete IGF-1 which is crucial for maintaining the pluripotency of spermatogonial stem cells and promoting their proliferation (277). In germ cells, IGF-1R contributes to their proliferation *via* the PI3K/AKT/mTOR/HIF2α pathway under hypoxic conditions (278). In Sertoli cells, IGF-1R can promote membrane depolarization and the cellular uptake of calcium, glucose, and amino acids *via* the PI3K/AKT pathway (271).

#### Ovary

As in the male gonad, IGF-1R plays a critical role in the function of ovarian cells, particularly granulosa and theca cells, in concert with FSH. These cells are essential for folliculogenesis, steroidogenesis, and overall ovarian function. *In vitro* studies on granulosa cells (GCs) have demonstrated that the stimulatory effect of FSH on aromatase expression, which is a marker of GC differentiation, relies on the action of IGF-1, and this process is dependent on the expression and activation of IGF-1R (279). GCs lacking IGF-1R are prone to apoptosis and exhibit a significant reduction in AKT activation and steroidogenic gene expression (280). These mice are viable and develop normally but are infertile. Their ovaries do not develop antral follicles and do not ovulate even after treatment with exogenous gonadotropins. In addition, GCs proliferation induced by FSH includes IRS-1, PI3K, and AKT (281).

IGF-1R activity is also important in theca cells, acting in part with the pituitary luteinizing hormone (LH) (282). IGF-1 is a potent stimulator of theca cell proliferation, increasing the number and proportion of steroidogenically active cells (282). IGF-1 stimulates the expression of steroidogenic enzymes CYP11A1 and 3βHSD in these cells (283) and of CYP17A1 only in the presence of LH (284). IGF-1 enhances both basal and LH-supported androgen biosynthesis in rat theca-interstitial cells (285). Androgens then diffuse to the granulosa layer where aromatase (CYP19A1), which is exclusively expressed in GCs, converts androgens into estradiol (286). GCs and theca cells cooperate to produce estradiol, which is critical for proliferation, follicle development, and the regulation of gonadotropin secretion (287). Thus, IGF-1 contributes to steroidogenesis in theca cells by both augmenting and mimicking LH actions.

#### Lung

This tissue is an important target of IGF-1, as mice lacking IGF-1R die of respiratory failure upon birth (288). Animal studies suggest that IGF-1, through its autocrine and paracrine effects (289), modulates the development and differentiation of various lung cell types, including airway basal cells, club cells, alveolar epithelial cells, and fibroblasts (290). Notably, the MAPK signaling pathway is activated during prenatal lung development (291). High expression levels of IGF-1R are observed in lung epithelial cells, alveolar macrophages and smooth muscle during different developmental stages. Indeed, the knockdown of IGF-1R disturbed airway epithelial differentiation in adult mice (292). In alveolar epithelial cells, IGF-IR modulates endocytosis through the PI3K/AKT pathway (197).

The GH-IGF-1 axis plays a crucial role in regulating the differentiation and proliferation of alveolar epithelial cells and neonatal lung fibroblasts (293). TGF-β1 derived from alveolar epithelial cells activates alveolar macrophages to secrete IGF-1 into the alveolar fluid in response to stimulation of the airway by inflammatory signals. Alveolar macrophage-derived IGF-1 attenuates the p38 MAPK inflammatory signal in alveolar epithelial cells and promotes the phagocytosis of apoptotic cells by alveolar epithelial cells (294). Under pathological conditions such as idiopathic pulmonary fibrosis (IPF), alveolar epithelial cells release IGF-1, which activates IGF-1R on adjacent normal alveolar epithelial cell surfaces, and further activates downstream PI3K and AKT kinases (252, 290, 295). Activation of the IGF-1R/PI3K/AKT pathway in the lung of IPF patients is associated with the upregulation of core fucosylation (CF) levels in senescent alveolar epithelial cells. Downregulation of CF prevented IGF-1-induced senescence in alveolar epithelial cells by inhibiting the activity of the IGF-1R/PI3K/AKT signaling pathway (198). Furthermore, activation of the IGF-1/IGF-1R axis led to the generation of reactive oxygen species (ROS), which promotes epithelial-mesenchymal transition (EMT) in alveolar epithelial cells, thereby accelerating pulmonary fibrosis (296). Similarly, IGF-1R deficiency in mice confers protection against alveolar damage and pulmonary inflammation (297).

Additionally, IGF-1 promotes the proliferation of pulmonary fibroblasts through IGF-1R. This leads to the upregulation of the Fos proto-oncogene activator protein one transcription factor subunit, as well as early growth response proteins EGR1 and EGR2 (207). In lung macrophages, IGF-1 levels were significantly elevated in asthmatic mice, with most of it coming from alveolar macrophages (AMs) (298). In models of acute lung injury (ALI) induced by lipopolysaccharide, the production of IGF-1 in the lungs increases significantly. This increase is primarily attributed to AMs, which secrete IGF-1 into the alveolar fluid (299). These findings suggest that IGF-1 derived from AMs may play a crucial role in regulating airway inflammation and remodeling. A recent study elucidated the mechanism of IGF-1 release through macrophages and its role in regulating inflammation. Transforming growth factor-β1 derived from alveolar epithelial cells (AECs) activates AMs to secrete IGF-1 into the alveolar fluid in response to inflammatory signals in the airway. This IGF-1 from AMs reduces the p38 MAPK inflammatory signal in AECs and enhances the phagocytosis of apoptotic cells by AECs (294). Overall, IGF-1 plays a multifaceted role in lung health, particularly through its interactions with macrophages and epithelial cells to regulate inflammation and promote tissue repair.

#### Pancreas

During the prenatal period, the differentiation and maturation of many tissues, including pancreatic β-cells, rely on the interaction of IGF-1 and IGF-2. These growth factors, which are essential for prenatal development, exert their actions through binding to their respective receptors (300). In the diabetic Goto–Kakizaki rat model, defective IGF-2 and IGF-1R protein production in the embryonic pancreas precedes β-cell mass anomaly (301). However, studies in mice with β-cell-specific knockout of the IGF-1R revealed that these mice exhibited normal growth and β-cell development, although they had defective glucose-stimulated insulin secretion and impaired glucose tolerance. These findings suggest that IGF-1R in mice may not be essential for islet cell development, but plays a significant role in β-cell function (302). IGF-1R in the pancreas is also involved in the regulation of cell growth. Hence, even though insulin-secreting β-cells are highly differentiated in adult humans, they retain the ability to proliferate (303). IGF-1R activity promotes β-cell proliferation through the activation of IRS-2 (304). The involvement of the PI3K pathway in the survival of pancreatic β-cells, along with the positive effect of MAPK activation on gene transcription, demonstrates the interconnected roles of the IR and IGF-1R in promoting β-cell growth (303). Indeed, IGF-1R promotes insulin secretion in pancreatic β cells in gestational diabetes mellitus (305). Moreover, serum IGF-1 levels have been confirmed to intensify insulin sensitivity and preserve the proper functioning of pancreatic β-cells (306). Conversely, the specific role of IGF-1R in α pancreatic cells, which secrete glucagon, remains largely unexplored. This represents a significant gap in our understanding of pancreatic endocrine function and offers a promising new area of research, as understanding how IGF-1R influences alpha pancreatic cells could provide new insights into the regulation of blood glucose levels and the pathophysiology of diabetes.

#### Skin

Several studies have demonstrated the role of IGF-1R and its signaling components in the skin. Both dermal fibroblasts and epidermal keratinocytes (a type of epithelial cell in the epidermis) express IGF-1R. When stimulated by IGF-1, these cells exhibit increased proliferation and mitogenic activity (307, 308). Higher levels of IGF-1 or IGF-1R are linked to enhanced cell proliferation, skin hyperplasia, and tumor formation (309). IGF-1/IGF-1R signaling is particularly crucial during development. IGF-1 knockout (KO) mice, which die immediately after birth, display skin abnormalities, including a thinner and disrupted epidermis with impaired barrier function and defective hair formation (288). Furthermore, mice with an epidermis-specific conditional IGF-1R KO have a thinner epidermis, indicating that IGF-1/IGF-1R signaling is essential for maintaining epidermal basal cells (310). Additionally, IGF-1 signaling may impact the human epidermis (311), as patients with conditions involving hyposecretion, such as Laron syndrome (characterized by primary IGF-1 deficiency and growth hormone resistance), are not only short in stature (312) but also exhibit early aging phenotypes like wrinkles (313).

#### Kidney

IGF-1R is necessary for normal kidney development (314), including podocyte cell survival and integrity of the glomerular filtrating barrier, playing a crucial role in maintaining normal tubular and glomerular functions through the PI3K/AKT and MAPK pathways (315). Research on IGF-1 expression during mouse kidney development has shown that IGF-1 mRNA is present in all embryonic kidney cells but significantly reduces its expression postnatally (316). In early embryonic stages, IGF-1R mRNA is expressed in the rat mesonephros and continues to be found in all nephron segments into adulthood (21). In human kidneys, IGF-1R is highly expressed in the glomeruli and tubular epithelial cells (317). IGF-1 knockout mice have proportionally small kidneys with decreased glomerular size and nephron number (318). Administration of IGF-1 has been shown to enhance renal plasma flow and glomerular filtration rate (GFR) (319). It impacts the GFR and blood flow of individual nephrons by increasing the ultrafiltration coefficient and reducing resistance in the efferent arterioles (320). This effect is mediated by the production of endogenous vasodilators, such as nitric oxide (NO) and prostaglandins, (321). The tandem IGF-1/IGF-1R directly enhances phosphate reabsorption by upregulating Na-Pi2a expression in the proximal tubule (322).

Podocytes preserve the integrity of the kidney's glomerular filtration barrier (323). Current evidence suggests both deleterious and beneficial effects of IGF-1R in this context (315, 324, 325). IGF signaling, essential for the survival of these cells, operates through the IGF-1R receptor *via* PI3K and MAPK pathways (315). However, in the context of high glucose conditions, IGF-1 might contribute to adverse cellular effects, including podocyte injury and apoptosis. Knocking down IGF-1 decreased IGF-1R levels, which alleviates high glucose-induced podocyte injury and apoptosis by inactivating the JAK2/STAT signaling pathway and enhancing autophagy (326). Transgenic mice with podocyte-specific dominant negative IGF-1R exhibit abnormal, small glomeruli and show signs of podocyte foot process effacement (327). A recent study showed that near total loss of murine podocyte IGF-1R is detrimental, which results in mitochondrial dysfunction, while partial inhibition protects from doxorubicin-induced podocyte injury through protection from oxidative stress (328).

In glomerular mesangial cells, IGF-1 stimulates cell-cycle progression, hypertrophy, and ECM protein synthesis through the AKT/GSK-3β, p38MAPK, and calcineurin pathways (329). Activation of IGF-1R safeguards both normal human mesangial cells and immortalized murine mesangial cell lines (MMC) from apoptosis triggered by glycol-oxidant stress. This protective anti-apoptotic effect of IGF-1R relies on the activation of both AKT and MAPK pathways (330). Similarly, activated IGF-1R was found to protect MMC and normal human mesangial cells from hyperglycemia-induced DNA damage, inducing a strong oxidant-resistant phenotype, and inhibiting ROS production (331). Under pathological conditions, activation of IGF-1R signaling in mesangial cells might lead to excessive expression of hyaluronan synthase 2 (Has2), a key enzyme involved in the synthesis of hyaluronan, a component of the extracellular matrix. This could contribute to pathological extracellular matrix accumulation, fibrosis, and altered tissue architecture, ultimately impairing kidney function. Genetic deletion of IGF-1R, but not IR, in the mesangial cells (MCs) inhibited both IGF-1 and insulin-induced expressions of Has2. IGF-1-induced phosphorylation of IGF-1R, AKT, and MAPK were significantly inhibited in both IR or IGF-1R-deleted MCs. IGF-1 signaling *via* IGF-1R on MCs may contribute to the pathogenesis of diabetic kidney disease through the induction of Has2 and fibrotic genes (332).

#### Spleen

IGF-1 produced by macrophages and other immune cells in the spleen in response to various stimuli, including cytokines and growth factors, plays a critical role in growth rate and erythropoiesis -the process by which red blood cells are produced, contributing to the overall health and functionality of this organ through the PI3K/AKT/mTOR pathway (333). In addition, GH influences splenic growth and development through the local expression of IGF-1 (334). Within the spleen, macrophages create structures known as erythroblastic islands, which serve as niches where red blood cell precursors, or erythroblasts, undergo maturation. IGF-1 secreted by these macrophages delivers essential growth factors that are crucial for the proliferation and differentiation of erythroblasts (335). However, it remains unclear whether IGF-1 signaling through macrophages in the spleen directly regulates immune function, inflammation, and tissue repair. Further studies are needed to elucidate these mechanisms.

#### Immune system

The role of IGF-1R in this system is, in our view, largely neglected as compared to other systems, as it probably is a key regulator of its function. IGF-1 and IGF-1R are expressed in the thymus during both fetal and postnatal life, with IGF-1-mediated signaling playing a key role in the early stages of T cell differentiation during fetal development (14, 336), as well as in the proliferation of mature T cells (337). This receptor activates JNKs and PI3K pathways and contributes to normal T-cell survival (338), and the production of cytokines by circulating monocytes (339).

However, research on the physiological role of IGF-1R in B lymphocytes, which are pivotal to the adaptive immune system, is notably sparse. There are no comprehensive studies either on the effects of IGF-1R knockdown specifically in B cells. Investigation into the activation of signaling cascades such as PI3K/AKT, MAPK, and JAK/STAT pathways following IGF-1R stimulation in B cells is pending. Encouraging targeted research in this domain is essential for advancing our knowledge and potential clinical applications.

#### Brain

The presence of IGF-1R in brain cells was described long ago, showing cell-specific glycosylation characteristics of uncertain functional significance. This organ expresses one of the highest levels of IGF-1R in the body and is probably one of the best-studied targets of IGF-1. We will illustrate the variety of actions and pathways where brain IGF-1R is involved, without attempting a comprehensive review, as many abound (340, 341, 342).

In astrocytes, IGF-1 modulates glucose uptake recruiting MAPK and AKT signaling to regulate LRP1 association to Glucose transporter 1 (114, 115) and proteostasis through ample transcriptional regulation (343), using as yet uncharacterized upstream pathways. In neurons, the role of IGF-1R depends on neuron type and is time- (344) and concentration (345) dependent, two recently described traits that may be of great relevance in understanding the physiological significance of IGF-1. Neuronal IGF-1R activity modulates a wide repertoire of functions, but its pro-survival actions through the PI3K/AKT pathway are the best characterized (346). IGF-1 has also been reported to increase MCT2 expression in neurons through AKT, promoting the uptake of lactate (347) in a mechanism that probably works in concert with the promotion by IGF-1 of lactate production in astrocytes (115). Modulation of Ca^2+^ entrance in cerebellar granule cells is mediated by Src phosphorylation of the αC1 subunit of the Ca^2+^ L channel (348) or AKT activation (349), whereas modulation of this channel subunit in hippocampal/cortical neurons involves phospholipase C, calcium release from IP_3_-sensitive internal stores, and calcium/calmodulin kinase II (350), suggesting an exquisite specificity of IGF-1R activity for each type of neuron. However, signaling pathways modulating other numerous ion currents in neurons by IGF-1R require further study. In adult neural progenitor cells, IGF-1R is mitogenic through MAPK (351). In oligodendrocytes, IGF-1R, acting *via* IRS-2, plays a crucial role in regulating the timing of myelination (352) and promotes oligodendrocyte proliferation through MAPK/Creb/Cyclin D1 (353). In remyelination processes, IGF-IR activity is also involved (354), in part through a subset of neonatal microglia (355), but in this case, underlying pathways have not been reported. Microglia, a key cell in immune surveillance, phagocytosis of tissue debris, and development, is an important producer of IGF-1 in the brain (355). Although in the *Drosophila* brain, ILP signaling through AKT is involved in cell debris phagocytosis by ensheathing glial cells that play a similar role to microglia (356), in vertebrate microglia, the role of IGF-1R in the healthy brain is not well studied.

## IGF-1R activity in pathology

IGF-1R plays a significant role in various pathological events, consistent with its wide range of actions in healthy states. While it likely contributes to many pathological processes, a comprehensive evaluation of its role is beyond the scope of this work, as numerous disease-specific reviews are available. Here, we will focus on two major groups of pathologies—cancer and neurodegeneration—where the majority of information is concentrated, and briefly address other significant conditions. Since IGF-1R is present in all tissues, its role in many diseases remains insufficiently explored. However, in certain pathologies involving specific cells or tissues, or in cases of known mutations affecting IGF-1R activity or its ligands, its role is more clearly established, as seen in thyroid eye disease (357), and Laron's dwarfism (358).

Pathology is perhaps the best example of the context-dependent roles of IGF-1R. For instance, in cancer, the role of IGF-1R is highly dependent on the cancer type, specifically which tissues and cells are affected. In neurodegenerative diseases, the situation is similar, with both beneficial and detrimental actions of IGF-1R documented (see below). A key aspect that is often overlooked in relation to the role of IGF-1R in pathology is the stage at which its role is examined, as disease progression determines tissue and cell changes that will inevitably be reflected in IGF-1R activity. We will address this crucial topic whenever information is available.

### Cancer

**Figure 5:**
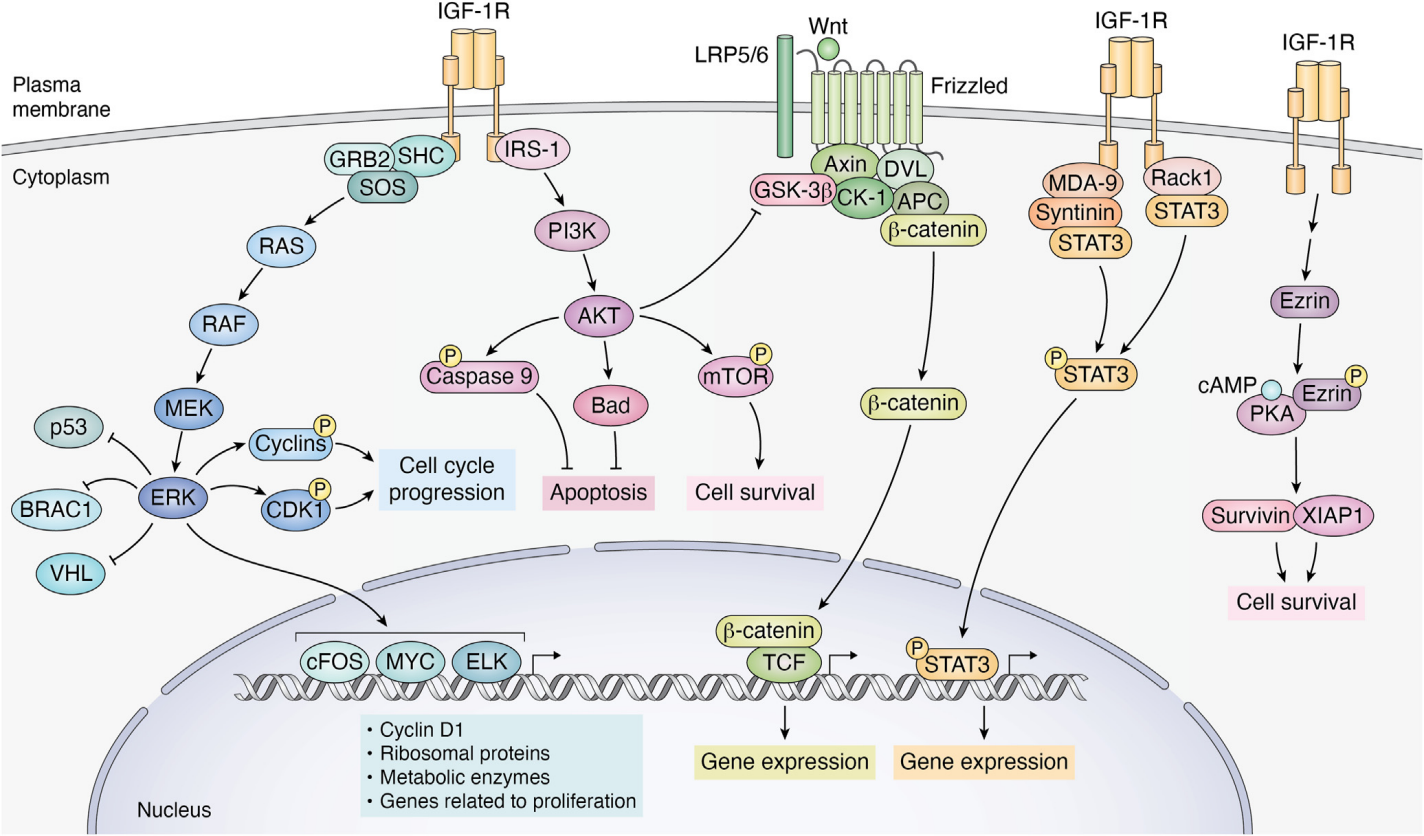
**Complexity of Signaling Pathways Involving IGF-1R in Pathology.** IGF-1R signaling in pathology also encompasses both canonical and non-canonical pathways. Canonically, IGF-1R activates the Ras/MAPK pathway, leading to ERK activation. ERK blocks tumor suppressors such as p53, BRAC1, or VHL (Von Hippel-Lindau) and phosphorylates cell cycle regulators like cyclins and CDKs, as well as transcription factors that control the expression of cyclins, ribosomal proteins, metabolic enzymes, and genes related to proliferation. The IGF-1R/IRS-1/PI3K/AKT pathway phosphorylates key apoptotic proteins such as caspase 9 and/or BAD, inhibiting them, while also activating mTOR *via* phosphorylation. This leads to the inhibition of apoptosis and the promotion of cell survival. Additionally, IGF-1R, through the IRS-1/PI3K/AKT pathway, inhibits GSK3β by phosphorylation. This action disrupts the DVL, Axin, APC, CK1, and β-catenin complex associated with Wnt signaling, allowing β-catenin to translocate to the nucleus. In the nucleus, β-catenin associates with TCF to activate the transcription of genes associated with epithelial-mesenchymal transition (EMT). Furthermore, IGF-1R can recruit STAT3 for phosphorylation through adapter proteins like Rack1 and/or the MDA-9/syntenin complex. Once phosphorylated, STAT3 translocates to the nucleus to activate the transcription of target genes. IGF-1R also controls the actin cytoskeleton *via* Ezrin, which recruits active PKA. This complex, though not fully understood, regulates cell survival through survivin and XIAP1.

This pathology represents the single most studied topic in relation to IGF-1R signaling pathways, probably because it was suggested in early studies a key role of IGF-1R in tumorigenesis (359). As a result, most studies describing intracellular IGF-1R signaling have been conducted in tumor cells (Fig. 5), which poses a cautionary note when translating these observations to their healthy counterparts. Importantly, after the repeated failures in exploiting the therapeutic potential of IGF-1R antagonists (360), the study of IGF-1R activity has lost momentum. As a result, only fragmentary knowledge of many IGF-1R pathways, not only in cancer, is available.

IGF-1R plays a critical role in tumor progression and has been extensively documented in numerous cancer types, including breast, ovary, prostate, colorectal, pancreatic, melanoma, and thyroid (361). Canonical PI3K/AKT pathway promotes cell survival and growth, involving mechanisms that prevent apoptosis, which is programmed cell death (49). Apoptosis is a controlled process crucial for maintaining cellular homeostasis and eliminating damaged or unnecessary cells. In this particular case, activation of IGF-1R triggers downstream signaling events that lead to the phosphorylation and activation of proteins involved in cell survival. As commented above in canonical signaling, this pathway involves recruitment by IGF-1R of the docking proteins Shc and IRSs upon its auto-phosphorylation in response to IGF-1 binding. Recruitment of these docking proteins not only promotes the membrane retention of IGF-1R but also mediates the activation of downstream signaling pathways of the IGF axis, which are primarily the PI3K/AKT and Ras/MAPK pathways. These two canonical pathways activate context- and cell-dependent targets that most commonly include GSK-3/Foxo/mTOR downstream of AKT -usually associated with cyto-protection, and Elk1/CREB/c-Myc transcription factors, downstream of MAPK and usually associated with cell cycle and migration/protein synthesis (Fig. 1).

Examples of the involvement of canonical IGF-1R activity include the observation that specific inhibitors targeting the MAPK and PI3K pathways reduced IGF-1-induced invasion ability of prostate cancer cells (362), or that IGF-1, primarily functioning through the PI3K/AKT/mTORC1 pathway, promotes the growth of preneoplastic prostate epithelial cells by counteracting autocrine TGF-β suppression of Survivin transcription (363). The IGF network is associated with normal epithelial differentiation and maintaining tissue homeostasis in the prostate (364, 365). Noncancerous prostate cells and prostate cancer cells respond differently to IGF exposure and IGF-1R overexpression. In cancer cells, IGFs boost proliferation. Conversely, in noncancerous epithelial cells, these signals promote basal to luminal differentiation. Therefore, IGFs act as growth factors for cancer cells but induce differentiation in non-cancerous prostate epithelial cells (366). In turn, elevated plasma levels of IGF-I have been shown to predict the incidence and stage of prostate cancer (PCa) (367). Notably, transgenic mice overexpressing IGF-1 develop PCa (368), and neutralizing antibodies against IGF-1R inhibit the growth of PCa xenografts (369). *In vitro*, overexpression of IGF-1R in prostate cancer cell lines led to increased cell proliferation, colony formation, migration, invasion, and resistance to apoptosis, while downregulation of IGF-1R produced the opposite effects. Interestingly, in non-cancerous prostate epithelial cell lines, overexpression of IGF-1R inhibited these cells, while knockdown of IGF-1R stimulated their activity (366, 370).

In pancreatic tumors, activation of IGF-1R stimulates the PI3K/AKT and Ras/Raf/MAPK signaling pathways negatively impacting tumor suppressor proteins such as p53, breast cancer 1 (BRCA1), and von-Hippel Lindau (VHL) (371, 372). Considerable attention has been directed towards understanding the implications of IGF-1R in pancreatic tumors that originate from epithelial cells. These tumors primarily include pancreatic ductal adenocarcinoma (PDAC) and various cystic neoplasms. In patients with PDAC, research has indicated elevated levels of IGF-1R expression within tumors (373), which has been linked to more advanced tumor grades and poorer survival outcomes (374). Additionally, increased concentrations of IGF-1 and IGFBPs have been identified in the blood and tissues of PDAC patients (375). *In vitro* experiments demonstrated that the addition of IGF-1 promotes the growth of PDAC cancer cell lines (376). This growth-stimulating effect can be inhibited by using an antibody specific to IGF-1R (377).

Colonic adenocarcinomas originate in the epithelial cells of the colon. Both IGF receptors are expressed in normal colonic epithelial cells and colorectal cell lines (378, 379). High concentrations of IGF-1 and -2 have been demonstrated in human colonic adenocarcinomas and exert mitogenic effects through paracrine/autocrine interactions with IGF-1R (380). This receptor is expressed at a high level in the adenoma stage, suggesting that the abnormal epithelia in adenomatous polyps may develop the capability to utilize IGF-1 from the surrounding environment more efficiently than normal colorectal epithelia. Overexpression of IGF-1R may be a molecular event associated with the precancerous transition of colorectal epithelial cells (381).

Lung adenocarcinoma cells are derived from the epithelial cells lining the small air sacs (alveoli) in the lungs. IGF-1R is currently being evaluated as a pharmacological target in clinical trials (382), including non-small-cell lung cancer (NSCLC). Elevated amplification and mRNA expression of IGF-1R, along with increased protein levels of IGF-1R and phosphorylated-IGF-1R in tumor tissues and serum samples from NSCLC patients was observed. Additionally, mice deficient in IGF-1R exhibited reduced tumor growth, cell proliferation, inflammation, and vascularization, and showed enhanced apoptosis after heterotopic tumor transplantation (383).

The importance of IGF-1R signaling in epithelial carcinogenesis is evidenced by the fact that IGF-1/IGF-1R are highly expressed in various cancers, including skin cancer, where inhibiting IGF-1R production has been shown to improve its outcome by reducing the activation of tumor invasion markers associated with skin cancer (384). Elevated PI3K/AKT activity and subsequent activation of one or more downstream effector pathways contributed significantly to the tumor-promoting action of IGF-1 in the epidermis of BK5 (Keratin 5, derived from epithelial cells), a skin cancer model. Topical application of the PI3K inhibitor LY294002 directly inhibited the persistent biochemical changes observed in the epidermis of BK5 (385). A reduction in circulating levels of IGF-1 can significantly inhibit the development of skin tumors in mice (386). This substantial decrease in susceptibility to skin tumor development is associated with reduced signaling through IGF-1R and the PI3K/AKT pathway (387).

Beyond these canonical signaling cascades, IGF-1R can also activate non-canonical signaling pathways (Fig. 5). For instance, Zong *et al.* demonstrated that STAT3 is one of the downstream targets of IGF-1R, mediating the phosphorylation of STAT3 at Tyr705 (388). A role of IGF–1R–mediated STAT3 activation has been reported in pancreatic and prostate cancer and melanoma (389). However, it is not clear how IGF-1R controls STAT3 activation in cancer. Zhang *et al.* have reported that IGF-1R relies on the adaptor protein RACK1 to recruit STAT3 for tyrosine phosphorylation (390). Another adaptor protein that could participate in STAT3 activation mediated by IGF-1R is MDA-9/Syntenin. However, the controversy in this case centers on MDA-9/Syntenin, as it appears to control invasion rather than participate in proliferation (391) Another non-canonical pathway involves the kinase PKA, which participates in IGF-1 signaling in colon cancer (392).

Conversely, recent evidence strongly indicates that the IGF-1/IGF-1R signaling pathway plays a significant role in promoting tumor metastasis and drug resistance associated with epithelial-to-mesenchymal transition (EMT) (393, 394). In this regard, the association between canonical Wnt/β-catenin signaling and IGF-1R signaling may contribute to the EMT process (395) In fact, in human colon cancer cells, IGF-1R promotes β-catenin translocation and stability by inhibiting GSK-3β, enhancing cell motility and facilitating colon cancer metastasis (396). Furthermore, IGF-1R cooperates with the Wnt signaling pathway in metastasis by activating TCF/LEF-dependent transcription through the AKT/GSK-3β/β-catenin pathway (397). In lung adenocarcinoma cells, IGF-1R-mediated EMT is probably dependent on the STAT3 and STAT5 pathways (398, 399). *TROP2* gene, that codes a cell surface glycoprotein is downregulated in lung adenocarcinoma patients, and its silencing through DNA methylation leads to a decreased suppression of IGF-1R signaling. This may lead to cancer progression (*e.g.* invasion, metastasis, and/or angiogenesis) *via* activation of IGF-1R signaling and its downstream mediators β-catenin and slug (400). In breast cancer, IGF-1 triggers the STAT3-dependent transcriptional activation of the S100A7 gene through IGF-1R in adjacent endothelial cells and contributes to angiogenesis (401). Increased angiogenesis is required for oxygen and nutrient delivery to enlarging solid tumors (402), and it enhances metastasis by facilitating cancer cell entry into abnormal, tumor-induced vessels (403). Angiogenesis inhibitors are in clinical use for the treatment of several advanced cancers.

Metastatic cancer cells exhibit characteristics such as unchecked cell growth and survival, decreased cell-cell adhesion, heightened ability to degrade the extracellular matrix, and increased cell motility. Changes in cell motility involve cytoskeleton reorganization and activation of multiple signaling pathways, which can be stimulated by growth factors. IGF-1 has been identified as playing a crucial role in enhancing the motility of breast and colon cancer cells (404). In this regard, the small GTPases of the Rho family comprise a group of signaling molecules that regulate a wide range of biological processes in cancer, including cytoskeleton re-arrangement, cell-cycle control, apoptosis, tumorigenesis, adhesion, invasion, and metastasis (405). The members of the small GTPases of the Rho family and their effector molecules are overexpressed in breast tumors, and their expression levels positively correlate with the progression of breast cancer (406). Small GTPase RhoC is overexpressed in 90% of inflammatory breast cancer and positively correlates with the tumor metastatic potential (407). In rats, small GTPases of the Rho family have been found to regulate mammary epithelial cell growth and metastasis (408).

The mechanical properties of the cell are largely regulated by the cytoskeletal architecture of microfilaments, intermediate filaments, and microtubules. Zhang *et al.* have shown that IGF-1R is unable to activate Rho in attached cells. However, in cell suspensions, IGF-1R rapidly stimulated RhoA activation and deactivated RhoB, while no changes in the levels of the active forms of RhoC, RhoG, or Cdc42 were detected (409). These findings suggest that IGF-1R-mediated RhoA activation may have a very important role in detached cells, such as circulating tumor cells (410). However, the detailed molecular mechanisms by which small Rho GTPases are activated remain unknown (49).

### Neurodegenerative diseases

Given the important role of ILPs in the brain (342), their pathological significance in brain disorders has been extensively studied and is evolutionarily conserved (411). Circulating levels of IGF-1 have even been proposed as a *“biomarker of risk stratification of brain health”* (412). Since revisions on this topic abound (413, 414), we will provide a general overview of IGF-1R signaling pathways described in the different types of conditions and brain cells involved.

#### Neuroinflammation

During inflammatory processes commonly associated with neuropathology, astrocytes and microglia experience morphological remodeling, -gliosis (415), that is regulated by IGF-1R (416). This receptor regulates phagocytosis and contributes to the resolution of neuroinflammation in both types of cells (160, 417). Another type of brain-resident immune cell is the border-associated macrophage (BAMs), which also plays a role in immune surveillance, waste clearance, nutrient uptake, and interaction with other patrolling cells (418). These cells secrete cytokines chemokines, lipokines, hormones, and growth factors to support inflammation or tissue regeneration through context-dependent immune modulation mechanisms (419). IGF-1 is locally secreted by microglia and BAMs in the central nervous system (CNS) and acts as a pro-remyelinating factor (420, 421). In the CNS, IGF-1R is expressed by virtually all resident and invading inflammatory cells, with IGF-1 in turn being able to modulate the function of different inflammatory players. Expression of IGF-1 was also observed in human microglia and macrophages and conferred strong protection against cytokine-mediated neuronal death in human fetal neuronal cultures (420). IGF-1 can even partly inhibit macrophage apoptosis by protecting mitochondria and decreasing macrophage invasion and activation of proinflammatory cytokines. Current studies suggest that IGF-1R signaling in BAMs/microglia exerts a significant protective role in limiting the development of CNS autoimmunity (422). However, the mechanism underlying this amelioration remains unclear.

#### Amyotrophic lateral sclerosis (ALS)

Based on evidence in animals (423) and humans (424), a key pathogenic role of IGF-1 in ALS was suggested (425), leading to the therapeutic use of this growth factor, first in animal models (425, 426), and then in clinical trials (427, 428). Since the results were not consistent (429), and experimental data did not always confirm therapeutic efficacy (430), IGF-1 therapy has not reached the clinic for ALS. Interference in IGF-1 availability by high local levels of IGFBPs may help explain this outcome (431). Indeed, pre-clinical and epidemiological findings continue to confirm a role for IGF-1 in ALS (432, 433). Recent evidence indicates that the mTOR pathway driven by IGF-1R in ALS astrocytes, which can rescue ALS motor neurons (434), is important in this neuropathology (435), but more information is needed.

#### Alzheimer's dementia (AD)

Among neurodegenerative diseases, AD is the best-characterized pathology in terms of the role of IGF-1R, where it seems to play an important, albeit somewhat controversial, role (436). Recent reviews provide an in-depth assessment of the role of IGF-1 signaling in AD (437, 438). Proteins involved in IGF-1R canonical signaling such as IRS-2 (439), AKT (440) or GSK-3β (441) have been involved in AD pathology, while reduced SUMOylation of IGF-1R attenuates neuroinflammation in AD mice (127). Further, phosphorylation of the amyloid precursor protein (APP) is modulated through PI3K/AKT by IGF-1R (442), and the IGF-1R/CaMKIV/Histone acetyltransferase pathway underlies environmental protection against AD features (443). The beneficial actions of IGF-1 on AD pathology (444, 445), not always confirmed (446), contrasts with the detrimental effects of IGF-1R (447, 448). However, the latter may be cell-type dependent (449, 450), which may relate to cell-specific IGF-1R pathways as PI3K/AKT are instrumental in neurons, while in astrocytes calcineurin appears to be involved in AD pathology (451).

#### Parkinson's disease (PD)

Clinical data hints to a role of IGF-1 activity in PD pathology (452). Accordingly, AKT activity appears to be involved in IGF-1 neuroprotection in experimental PD (453), and activity of this kinase in PD links PINK1, which is mutated in familial PD cases, with IGF-1R (107), while PD brains show increased levels of Ser-phosphorylated IRS-1, a marker of IGF-1R/IR insensitivity (454).

#### Huntington's disease (HD)

Toxicity of huntingtin, the protein mutated in HD, is decreased by IGF-1R activation *via* PI3K/AKT (455), while degradation of accumulated mutated protein is mediated by IRS-2 (456), providing a potential therapeutic target for this devastating disease.

### Metabolic diseases

IGF-1R plays an important role in metabolic and endocrine disorders because the tandem IGF-1/IGF-1R is critical in regulating glucose and lipid metabolism. Dysregulation of the IGF-1/IGF-1R axis is also associated with growth disorders such as acromegaly (457) and dwarfism (458), characterized by abnormal levels of IGF-1R.

While IGF-1R activity is involved in diabetes (459), a pathology affecting the whole organism, once more the involvement of IGF-1R pathways appears to be tissue-dependent. Additionally, since insulin and IGF-1 are functionally intertwined, the profound consequences of diabetes on insulin function heavily affect IGF-1/IGF-1R activity, and *vice versa*. However, pathways underlying the sensitizing role of IGF-IR on insulin (460), or its role in glucose metabolism need to be explored yet. Thus, deletion of muscle IGF-1R is sufficient to elicit type 2 diabetes (461), whereas low liver production of IGF-1 is also associated with type 2 diabetes (462), but responsible intracellular routes are unknown.

Although there is not much information available on IGF-1R-related pathways involved in diabetic pathology in key tissues such as skeletal muscle, liver, or adipocytes, a few examples can be provided. For instance, in the diabetic cardiac muscle, IGF-IR activity through ERK and AKT depends in the activity of the HSP60 chaperone and the mitochondria (463), and the latter pathway is attenuated in this cell type due to diabetes-associated inflammation (464). In the diabetic kidney, IGF-1R acts through AKT/GSK-3β/14-3-3 protein to promote the survival of kidney mesangial cells associated with renal hypertrophy (465). IGF-1R may also contribute to the pathogenesis of diabetic kidney disease through the induction of fibrotic genes and hyaluronan synthase 2, a key enzyme involved in the synthesis of hyaluronan, a component of the extracellular matrix (332). Wound macrophages collected from diabetic patients and mice show M1 macrophages have high levels of pro-inflammatory molecules such as TNF-α, interleukin (IL)-1 β, and matrix metalloproteinase 9, and relatively low levels of anti-inflammatory molecules and cytokines, such as TGF-β, IGF-1, and IL-10, typically associated with the repair phase (466, 467). Treatment with IGF-1 reversed diabetes effects and increased total hydroxyproline, DNA, protein, and macrophage in diabetes-induced wound healing impairment (468). IGF-1 helps to reduce the production of pro-inflammatory cytokines (*e.g.*, TNF-α, IL-1β) and increase the production of anti-inflammatory cytokines (168). This environment supports macrophage survival by minimizing cellular stress and damage associated with chronic inflammation.

Diabetes is also linked to various microvascular complications, including retinopathy. The role of IGF-1 in this condition is complex and challenging to understand. Some studies suggest that IGF-1 has a protective role, while others indicate it may be pathological (469). Increased levels of IGF-1 in vitreous have been correlated with the severity of diabetic retinal neovascularization (470). Conversely, some reports have shown that therapies targeting the IGF-I signaling pathway can prevent and reverse the development of diabetic retinopathy by regulating the AKT pathway. Studies have shown that various factors, including IGF-1, VEGF, and others, can regulate the AKT pathway. IGF-1 downregulation decreases the activation of AKT in diabetes retinopathy, which abrogates the neuroprotective effect, upregulates VEGF expression, and thus induces neovascularization (471). IGF-I treatment can prevent retinal cell death in diabetic rats and potentially help prevent neuronal cell loss in the retina in patients with diabetes (472, 473). IGF-1 also has been reported to be a potent stimulator of retinal endothelial cell growth and to play a major role in the development of diabetic retinopathy (192). Knockout of insulin and IGF-1 receptors on vascular endothelial cells protects against retinal neovascularization (181). However, one recent study highlighted the protective effect of IGF1 in combination with dopamine by downregulating VEGF. Anti-angiogenic activity of dopamine +IGF-1 was attenuated through ERK/AKT pathway (474).

## Open questions and future developments

Many open questions remain in the field. A major one is the role of IGF-1R in three key aspects; aging, neurodegeneration, and cancer, as both detrimental and beneficial actions have been documented. In aging, the search for possible genetic determinants using invertebrate models pointed to insulin peptides (475). These observations were confirmed in vertebrates (476), but not without controversy (477). Attempts to reconcile apparent contradictions abound (143, 478, 479), but no consensus has yet been reached. A similar controversy is present in age-associated neurodegenerative diseases, such as Alzheimer's disease, and others (436). Possible conceptual obstacles hindering clarification of the role of IGF-IR in aging and neurodegeneration are, for instance, equating high IGF-1 levels in blood with higher IGF-1 activity (480), not considering the ligand-independent actions of the receptor (140, 481), or the dose- and time-dependent effects of IGF-1 (344, 345). High serum IGF-1 likely reflects reduced IGF-1R sensitivity in aging (482, 483), similar to what was reported decades ago for the aged IR (484), with high insulin contributing to IR resistance (485). In addition, ligand-independent actions of IGF-1R, which are far from being well characterized, may confront ligand-dependent ones (96, 115). Reflecting this controversy is the fact that both inhibition (486), and sensitization (487) of IGF-IR have been reported to ameliorate age-associated conditions.

In cancer, since IGF-1 is a potent cytoprotective signal, IGF-1/IGF-1R activity has been long ago established as pro-tumorigenic (359). Encouraging pre-clinical observations with novel IGF-1R antagonists (488) and epidemiological surveys linking IGF-1 levels to cancer risk (489), led to many clinical trials with biologicals and small molecules, but with sobering results (360). The therapeutic failure of pharmacological IGF-1R blockade fueled in-depth analyses of underlying causes, providing a burst of novel insights in the modulation of IGF-1R signaling (490, 491). A review of ongoing oncological trials suggests that IGF-1R blockers are no longer used in cancer. Nevertheless, efforts to find new anti-cancer actions for these drugs continue (492, 493, 494), albeit at a lower pace. Importantly, anti-IGF-1R antibodies have turned out to be useful in Graves' disease (495). This positive outcome in this thyroid condition underscores our insufficient knowledge of IGF-1R in cancer.

Due to the association between IGF-1 and tumor growth, the therapeutic use of this growth factor has been approached with great caution. While IGF-1 is clinically approved to be used in IGF-1 deficiency, most prominently Laron's dwarfism (458), other clinical trials with encouraging results (496, 497, 498, 499, 500, 501) have not progressed to clinical application. Recently, a small molecule mimetic has been approved for Rett syndrome (502) as the result of previous successes with IGF-1 (503); however, further developments are not anticipated. We believe that the therapeutic potential of IGF-1/IGF-1R signaling remains very promising and should be further explored, as for example, in Down's syndrome (504). Finally, we also consider that the role of IGF-1R/IR hybrid receptors still needs thorough analysis, as they are found in all tissues and are modified in pathological conditions (505). However, novel tools should be developed and/or applied.

## Conclusions

In the majority of published studies, the characterization of the pathways downstream of IGF-1R is incomplete, focusing on early and/or late events. A key aspect of IGF-1 signaling is its context dependency (506) based on its wide expression in all types of cells, in every tissue along ontogeny, and throughout disease progression. While canonical IGF-1R signaling through MAPK and AKT is well established and probably constitutes the core of all actions of this receptor in target cells, multiple non-canonical and pseudo-canonical pathways are only partially characterized. Thus, despite intense scrutiny of IGF-1R activity during the last decades, we still have an incomplete picture of their signaling landscape. To help the field move forward, we consider that new studies using state-of-the-art tools such as CRISPR gene editing, advanced imaging (live-cell, super-resolution, bio-sensing), and single-cell proteomics/genomics combined with AI should be systematically applied in healthy and diseased tissues were IGF-1/IGF-1R actions are decisive.

## Conflict of interest

The authors declare that they have no conflicts of interest with the contents of this article.
